# Key role of lipid management in nitrogen and aroma metabolism in an evolved wine yeast strain

**DOI:** 10.1186/s12934-016-0434-6

**Published:** 2016-02-09

**Authors:** Stéphanie Rollero, Jean-Roch Mouret, Isabelle Sanchez, Carole Camarasa, Anne Ortiz-Julien, Jean-Marie Sablayrolles, Sylvie Dequin

**Affiliations:** INRA, UMR1083, 34060 Montpellier, France; SupAgro, UMR1083, 34060 Montpellier, France; Universite Montpellier, UMR1083, 34060 Montpellier, France; Lallemand SAS, 31700 Blagnac, France

**Keywords:** Wine yeast, Adaptive evolution, On-line monitoring, Transcriptome, Aroma compounds, Nitrogen, Phytosterols

## Abstract

**Background:**

Fermentative aromas play a key role in the organoleptic profile of young wines. Their production depends both on yeast strain and fermentation conditions. A present-day trend in the wine industry consists in developing new strains with aromatic properties using adaptive evolution approaches. An evolved strain, Affinity™ ECA5, overproducing esters, was recently obtained. In this study, dynamics of nitrogen consumption and of the fermentative aroma synthesis of the evolved and its ancestral strains were compared and coupled with a transcriptomic analysis approach to better understand the metabolic reshaping of Affinity™ ECA5.

**Results:**

Nitrogen assimilation was different between the two strains, particularly amino acids transported by carriers regulated by nitrogen catabolite repression. We also observed differences in the kinetics of fermentative aroma production, especially in the bioconversion of higher alcohols into acetate esters. Finally, transcriptomic data showed that the enhanced bioconversion into acetate esters by the evolved strain was associated with the repression of genes involved in sterol biosynthesis rather than an enhanced expression of *ATF1* and *ATF2* (genes coding for the enzymes responsible for the synthesis of acetate esters from higher alcohols).

**Conclusions:**

An integrated approach to yeast metabolism—combining transcriptomic analyses and online monitoring data—showed differences between the two strains at different levels. Differences in nitrogen source consumption were observed suggesting modifications of NCR in the evolved strain. Moreover, the evolved strain showed a different way of managing the lipid source, which notably affected the production of acetate esters, likely because of a greater availability of acetyl-CoA for the evolved strain.

**Electronic supplementary material:**

The online version of this article (doi:10.1186/s12934-016-0434-6) contains supplementary material, which is available to authorized users.

## Background

In a market becoming increasingly competitive, optimizing the quality of wines, especially the organoleptic properties, is a major challenge for the winemaker. Wine aroma is one of the principal attributes determining the preferences of wine consumers [1, 2]. Most fruity aroma compounds, esters in particular, are produced by yeast during alcoholic fermentation. Strategies to optimize the synthesis of aroma compounds may rely on the control of fermentation conditions, particularly through the addition of nutrients (nitrogen sources, lipids, etc.). Another approach is the development of wine yeast strains with improved aroma characteristics.

Several studies have already assessed the influence of fermentation parameters (principally nitrogen addition and temperature) on the production of fermentative aromas [3–6]. The yeast strain can also greatly affect the final concentration of these volatile compounds [7–10]. It is also conceivable that new yeast strains with superior aromatic properties compared to those of available commercial wine yeasts can be developed.

Over the past decades, several strategies based on genetic engineering approaches have been extensively explored, resulting in the development of wine yeast strains with improved fermentation abilities and with the capacity to increase the organoleptic quality of wine [11–14]. Despite the success of these studies, the poor consumer acceptance of genetically modified organisms (GMO) is a major obstacle to the use of these strains for winemaking. Therefore, GMO-free strategies, such as adaptive evolution approaches, have become strategies of choice for improving wine yeast traits [15–17].

Adaptive evolution is based on maintaining yeast over a large number of generations under conditions in which a specific selective pressure is applied. This approach favors the emergence of genetic variations and can result in adaptive evolution of the yeast population and in the selection of evolved variants with desired phenotypes. Using this approach, we obtained an evolved wine yeast (Affinity™ ECA5) exhibiting marked changes in central carbon metabolism, particularly an increased flux through the pentose phosphate pathway [15]. This strain displays several novel traits that are potentially beneficial for winemaking [15, 18]. This strain produced, relative to the parental strain (Lalvin EC1118^®^), markedly lower volatile acidity but greater amounts of higher alcohols and esters; these characteristics make Affinity™ ECA5 an attractive strain for enhancing the organoleptic qualities of wine [18]. Using a recently developed online monitoring system, the kinetic profiles of the production of various fermentative aroma compounds by the evolved and ancestral strains, Affinity™ ECA5 and Lalvin EC1118^®^, were compared [19]. The high frequency of acquisition of online gas chromatography allows for the determination of kinetic parameters and the calculation of the rates of synthesis for fermentative aromas [4]. This innovative tool also makes it possible to determine the gas–liquid balances of aroma production to distinguish yeast metabolic synthesis from physicochemical effects [20]. Mouret et al. [19] highlighted differences in the chronology of synthesis of fermentative aromas between the two strains, suggesting that the regulation of the synthesis of these compounds in the evolved strain differs from that in the ancestral strain, making the strains interesting models for metabolic studies.

The concentration of assimilable nitrogen is well known to have a major effect on fermentative aroma production (reviewed in [21]). At low nitrogen content, a direct relationship between initial nitrogen content and higher alcohol concentration is observed, whereas an inverse relationship is found at moderate to high nitrogen contents [6, 22–24]. A simpler relationship exists between nitrogen concentration and synthesis of acetate and ethyl esters: An increase in initial nitrogen content is associated with an increase in ester production [25–28]. Conversely, the effect of phytosterols on the synthesis of these molecules has only recently been studied [29].

The objective of this study was to explain (i) the differences in the production of fermentative aromas between the evolved and its ancestral strains and (ii) the effect of nutrients on the strains’ volatile molecule synthesis. To elucidate the mechanisms underlying aroma overproduction by Affinity™ ECA5, we combined a kinetic analysis of assimilation of nitrogen compounds and of fermentative aroma production with a comparative transcriptomic study of the two strains.

## Results

### Relative performance of Affinity™ ECA5 compared to that of Lalvin EC1118^®^

The properties of Affinity™ ECA5 and of the ancestral strain Lalvin EC1118^®^ have been compared in previous studies [15, 18, 19], and major differences have been identified. In particular, the production of fermentative aromas was enhanced for the evolved strain [18]. In the present work, we sought to evaluate the robustness of this phenotype under different environmental conditions. Using a Box-Behnken design [29], we evaluated the combined effects of three environmental parameters: initial content of assimilable nitrogen, phytosterols and temperature, with broad ranges of variation for each factor.

**Fig. 1:**
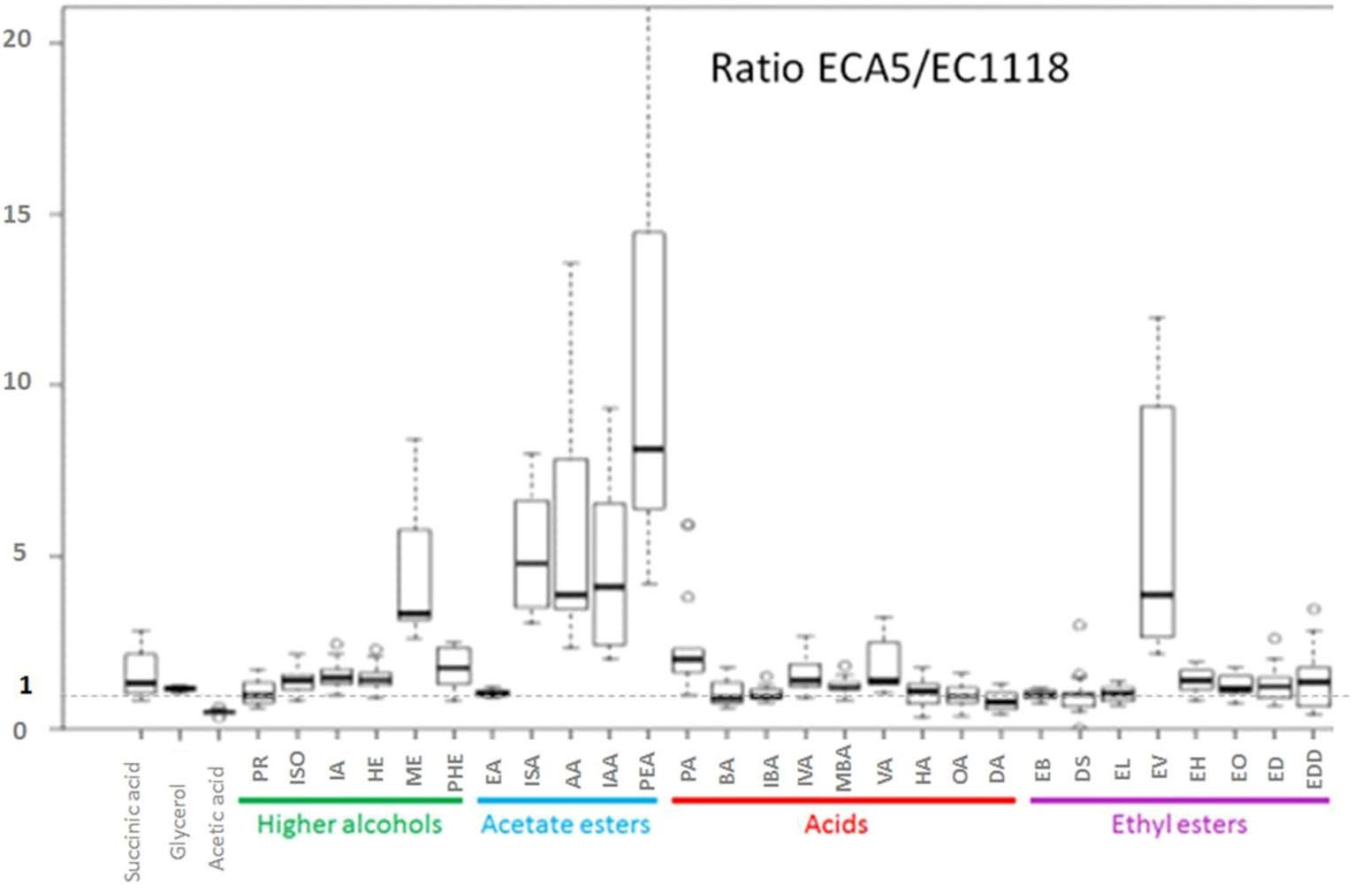
Ratios of final liquid concentrations of fermentative aromas produced by Affinity™ ECA5 and Lalvin EC1118^®^. These ratios were calculated from 16 fermentation experiments with different temperature, nitrogen and lipid contents. If the ratio value is higher than one, the compound is considered as overproduced by the evolved strain. *PR* propanol, *ISO* isobutanol, *IA* isoamyl alcohol, *HE* hexanol, *ME* methionol, *PHE* 2-phenylethanol, *EA* ethyl acetate, *ISA* isobutyl acetate, *AA* amyl acetate, *IAA* isoamyl acetate, *PEA* 2-phenylethylacetate, *PA* propanoic acid, *BA* butanoic acid, *IBA* isobutanoic acid, *IVA* isovaleric acid, *MBA* 2-methylbutanoic acid, *VA* valeric acid, *HA* hexanoic acid, *OA* octanoic acid, *DA* decanoic acid, *EB* ethyl butanoate, *DS* diethyl succinate, *EL* ethyl lactate, *EV* ethyl valerate, *EH* ethyl hexanoate, *EO* ethyl octanoate, *ED* ethyl decanoate, *EDD* ethyl dodecanoate

For each aroma compound, we plotted the ratio of the final concentration obtained with the strain Affinity™ ECA5 to that obtained for Lalvin EC1118^®^ (Fig. 1). These ratios were systematically greater than one for higher alcohols (except for propanol) and acetate esters. Conversely, we found that for the ratios for the fatty acids and ethyl esters, one was included in the confidence interval. Therefore, we can conclude that regardless of the conditions of fermentation, Affinity™ ECA5 systematically overproduced higher alcohols (except propanol) and acetate esters compared to the ancestral strain, whereas under certain conditions, the production of acids and ethyl esters did not enable a distinction between the two strains.

To better understand the mechanisms responsible for these differences, the production of volatile compounds was monitored during fermentation using an online monitoring system [30] for both strains. We studied the effect of the initial concentrations of nitrogen and phytosterol, which are key grape must nutrients that strongly affect aroma compounds [29]. Two levels of nitrogen (70 and 330 mg/L) and phytosterol (2 and 8 mg/L) were tested. For all fermentations, nitrogen was exhausted at the end of the growth phase and sugars were exhausted at the end of fermentation.

#### Effect of nutrients on nitrogen metabolism

**Fig. 2:**
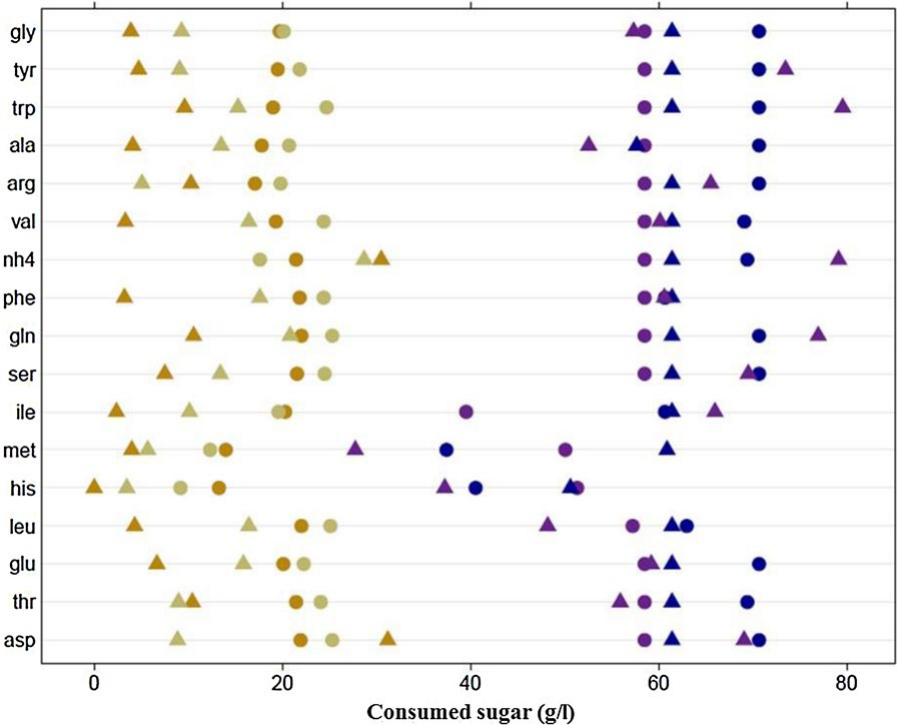
Timing of exhaustion of each nitrogen source (Point.AA0) expressed in terms of consumed sugar (g/L) for each fermentation condition. Lalvin EC1118^®^-SM70-2 mg/l of phytosterols (*closed orange circle*); Lalvin EC1118^®^-SM70-8 mg/l of phytosterols (*closed beige circle*); Lalvin EC1118^®^-SM330-2 mg/l of phytosterols (*closed blue circle*); Lalvin EC1118^®^-SM330-8 mg/l of phytosterols (*closed purple circle*); Affinity™ ECA5-SM70-2 mg/l of phytosterols (*closed orange triangle*); Affinity™ ECA5-SM70-8 mg/l of phytosterols (*closed beige triangle*); Affinity™ ECA5-SM330-2 mg/l of phytosterols (*closed blue triangle*); Affinity™ ECA5-SM330-8 mg/l of phytotserols (*closed purple triangle*). *Gly* glycine, *Tyr* tyrosine, *Trp* Tryptophan, *Ala* alanine, *Arg* arginine, *Val* valine, *NH4* ammonium, *Phe* phenylalanine, *Gln* glutamine, *Ser* serine, *Ile* isoleucine, *Met* methionine, *His* histidine, *Leu* leucine, *Glu* glutamate, *Thr* threonine, *Asp* aspartate

The consumption of nitrogen sources (amino acids and ammonium) was monitored throughout the cell growth phase until nitrogen exhaustion. Modeling this consumption allowed for the determination of the timing (expressed in consumed sugars) of depletion of each nitrogen source (called Point.AA0) for the different culture media and strains (Fig. 2).

As expected, the overall consumption phase of nitrogen was shorter in the media containing 70 mg/L of nitrogen compared to those containing 330 mg/L. Moreover, the order in which nitrogen sources were assimilated was generally the same for the two strains and consistent with that described by [31].

With SM70 (shown in orange and beige in Fig. 2), the consumption of the nitrogen source by Affinity™ ECA5 (triangles) was generally faster than that observed for Lalvin EC1118^®^ (circles), except for ammonium (Fig. 2). The dose of phytosterols also affected the consumption of various nitrogen sources for the two yeast strains. The nitrogen sources were most rapidly depleted at the lowest lipid concentration (in orange), but the effect was higher for Affinity™ ECA5, especially for valine, phenylalanine, leucine. Indeed, the differences between the values of Point.AA0 obtained at 2 mg/L and 8 mg/L were greater for this strain than for the ancestral strain.

With SM330 (shown in blue and purple in Fig. 2), the dose of phytosterols still modulated nitrogen consumption. However, an increase in phytosterol content typically had the opposite effect on this consumption for the two strains. Indeed, Affinity™ ECA5 consumed nitrogen more rapidly when the must contained 2 mg/l of phytosterols (in blue), whereas Lalvin EC1118^®^ generally consumed nitrogen faster with 8 mg/l (in purple). This opposite effect of lipid content on the Point.AA0 values was particularly pronounced for tyrosine, tryptophan, glutamine and ammonium (Fig. 2).

#### Effect of nutrients on fermentative aromas

We then decided to compare the abilities of the strains to synthesize fermentative aromas. Indeed, some amino acids are precursors of higher alcohols and acetate esters [32]. Therefore, we asked whether the observed differences in nitrogen assimilation induced certain variations in the production of volatile compounds.

We compared the kinetic profiles of production of fermentative aromas of the evolved and ancestral strains Affinity™ ECA5 and Lalvin EC1118^®^ using an online GC system and performed gas–liquid balances [4, 20] by differentiating between accumulation in the liquid phase, losses in the gas phase and total production (sum of liquid content and gaseous losses). Herein, we present data for the total production of fermentative aromas, which reflects the true capacity of the yeast to synthesize these volatile compounds. In addition, the high measurement frequency of this original device makes it possible to calculate the rates of total production of fermentative aromas [4, 33]. Biomass was also measured (off-line), allowing for the calculation of specific rates of production. Such rates represent metabolic fluxes and are essential for understanding yeast metabolism.

**Fig. 3:**
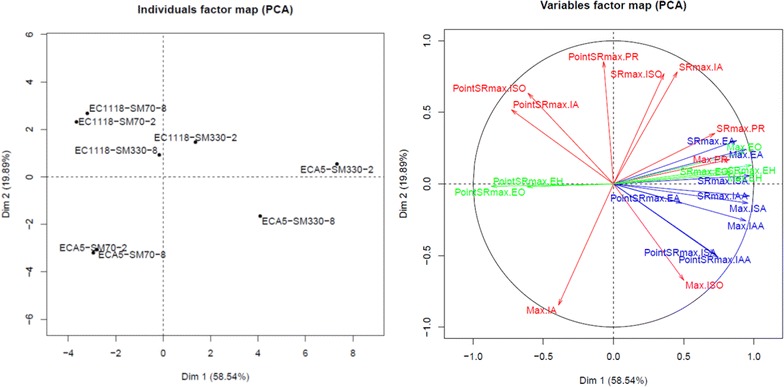
Principal component analysis (PCA) of the final production of volatile compounds (Max), the maximal specific rate of production (SRmax), and the time at which this maximum was reached (PointSRmax). Each fermentation is identified by the labels X, Y, and Z, where X corresponds to the strain, Y to the initial nitrogen concentration in mg N/l and Z is the phytosterol content in mg/l. *PR* propanol, *ISO* isobutanol, *IA* isoamyl alcohol, *EA* ethyl acetate, *ISO* isobutyl acetate, *IAA* isoamyl acetate, *EH* ethyl hexanoate, *EO* ethyl octanoate

With this dataset, we performed PCA using three parameters (maximum specific rate of total production: SRmax, sugar consumption at the maximal specific rate of production: PointSRmax and total production of each volatile compound: Max) to obtain an overview of the effect of fermentation conditions and strains on aroma synthesis (Fig. 3). The first two PCA axes accounted for 78.4 % of the total variation. The dispersion of fermentation conditions was greater for the fermentations performed with 330 mg/L of assimilable nitrogen and for the Affinity™ ECA5 strain. Another important finding was the differential effect of environmental changes depending on the class of studied compounds (higher alcohols, acetate or ethyl esters). For the acetate and ethyl esters, the effects of nutrients on SRmax and Max were similar. Indeed, the Max and SRmax of all esters were positively correlated and reached their maximum values for fermentations performed with Affinity™ ECA5 at high nitrogen content (Fig. 3, Table 1). By contrast, the chronology of synthesis (PointSRmax) of the esters was affected differently by changes in the environmental parameters for the two classes of esters. For acetate esters, PointSRmax reached its maximal value for fermentations performed using Affinity™ ECA5 at both high nitrogen and phytosterol levels. For ethyl esters, the highest value of this parameter was obtained in SM70 for both strains (Fig. 3). Finally, for ethyl esters there was the negative correlation between Max and SRmax, on the one hand and PointSRmax on the other hand (Fig. 3). Among the higher alcohols, propanol showed an atypical response. Its behavior was similar to that observed for the acetate esters (Fig. 3, Table 1). The SRmax and PointSRmax of isobutanol and isoamyl alcohol were similarly affected by the nutrient contents. However, these two higher alcohols differed in their total production: the maximal concentration of isobutanol was reached at both high nitrogen and phytosterol concentrations with Affinity™ ECA5; the maximal production of isoamyl alcohol was also obtained with the evolved strain but at a low nitrogen concentration (Fig. 3, Table 1). For isoamyl alcohol, a negative correlation between Max and SRmax was observed.

**Table 1:** Total production of fermentative aromas for each fermentation condition

Initial nitrogen concentration (mgIL)	Initial phytosterols concentration (mg/l)	Propanol		Isobutanol		Isoamyl alcohol	
		Total production (mg/l) by EC1118	Total production (mg/l) by ECA5	Total production (mg/l) by EC1118	Total production (mg/l) by ECA5	Total production (mg/l) by EC1118	Total production (mg/l) by ECA5
70	2	5.04	4.52	25.8	36.1	165	276
70	8	5.25	4.63	28.8	36.5	175	270
330	2	25.6	22.2	19.9	42.1	103	148
330	8	27.6	21.2	36.3	54.9	156	221

Initial nitrogen concentration (mg/l)	Initial phytosterols concentration (mg/l)	Ethyl acetate		Isobutyl acetate		Isoamyl acetate	
		Total production (mg/l) by EC1118	Total production (mg/l) by ECA5	Total production (mg/l) by EC1118	Total production (mg/l) by ECA5	Total production (mg/l) by EC1118	Total production (mg/l) by ECA5
70	2	37.7	27.1	0.05	0.23	0.45	3.29
70	8	39.7	22.9	0.05	0.19	0.43	2.14
330	2	67.7	124	0.36	1.79	3.83	13.0
330	8	52.8	73.3	0.31	1.45	2.87	12.0

Initial nitrogen concentration (mg/l)	Initial phytosterols concentration (mg/l)	Ethyl hexanoate		Ethyl octanoate			
		Total production (mg/l) by EC1118	Total production (mg/l) by ECA5	Total production (mg/l) by EC1118	Total production (mg/l) by ECA5		
70	2	0.67	0.72	0.64	0.62		
70	8	0.63	0.68	0.57	0.55		
330	2	1.32	1.71	1.71	2.05		
330	8	0.88	1.07	1.26	1.18		

**Fig. 4:**
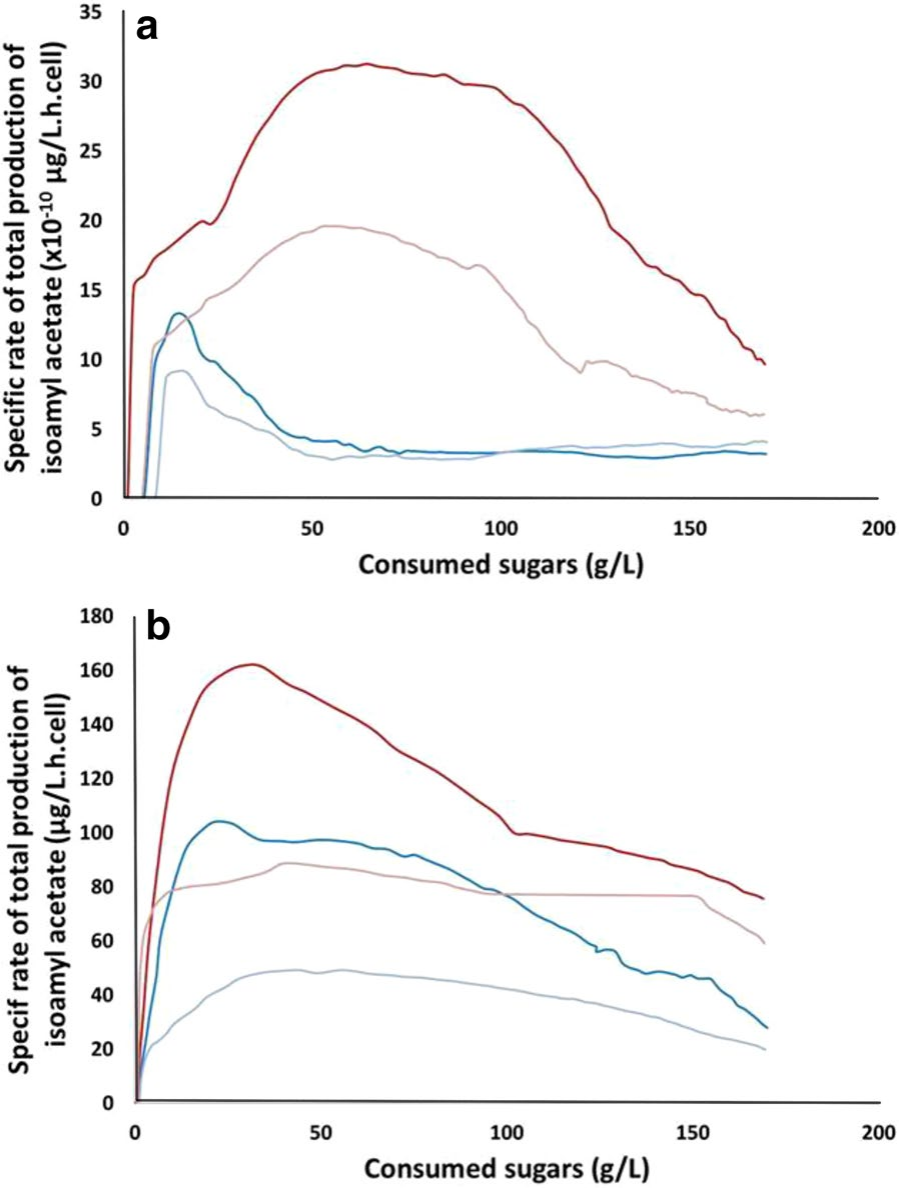
Changes in the specific rates of total production of isoamyl acetate in SM70 (**a**) or SM330 (**b**). Lalvin EC1118^®^−2 mg/L of phytosterol (*blue*); Lalvin EC1118^®^−8 mg/L of phytosterol (*light blue*); Affinity™ ECA5–2 mg/L of phytosterol (*red*); Affinity™ ECA5–8 mg/L of phytosterol (*pink*)

In addition to this general characterization, we performed a detailed analysis of the kinetic profiles of ester production, using isoamyl acetate as an example (Fig. 4). For SM330, we observed that the shape of the curve was related to the phytosterol content. At 2 mg/L of lipids, the specific production rate peaked very quickly and suddenly decreased, whereas at 8 mg/L of phytosterols, once the maximum value was reached the decrease was much slower (Fig. 4). Conversely, at low nitrogen content, the shape of the curve was mainly mediated by the yeast strain with a higher production observed for Affinity™ ECA5 than for Lalvin EC1118^®^. Finally, when comparing the maximal values of total production and of the specific rate of these esters, a striking difference was observed in the ranking of fermentation conditions according to these two parameters (Fig. 4, Table 1).

#### Relationship between nitrogen consumption and aroma production

We then studied the potential links between the consumption of amino acids and the production of fermentative aromas. For this purpose, two MFAs (one for each strain) were performed with the PointSRmax for volatile compounds and the Point.AA0 for the amino acid precursors of higher alcohols (leucine, valine, and threonine), ammonium and total assimilable nitrogen (Additional file 1). For Lalvin EC1118^®^, the first two MFA axes accounted for 98.8 % of the total variation (Additional file 1a), whereas these axes accounted for 94.6 % of the total variation for Affinity™ ECA5 (Additional file 1b). The relationship between the parameters was related to the assimilation of amino acids, and those representatives of aroma synthesis were completely different between the two yeast strains (Additional file 1). For Lalvin^®^ EC1118, the two classes of variables were negatively correlated (except for isobutyl acetate), whereas for Affinity™ ECA5, most of the variables were positively correlated.

### Bioconversion between higher alcohol and its acetate

In previous studies and in the general screening performed in this study using the Box-Behnken design, we observed a systematic overproduction of higher alcohols and acetate esters by Affinity™ ECA5 (Fig. 1). We considered whether the overproduction of acetate esters by the evolved strain was solely due to the overproduction of their higher alcohols and/or acetyl-CoA precursors or whether the activity of the alcohol acetyltransferases (Atf1p and/or Atf2p) responsible for this bioconversion was also involved.

**Fig. 5:**
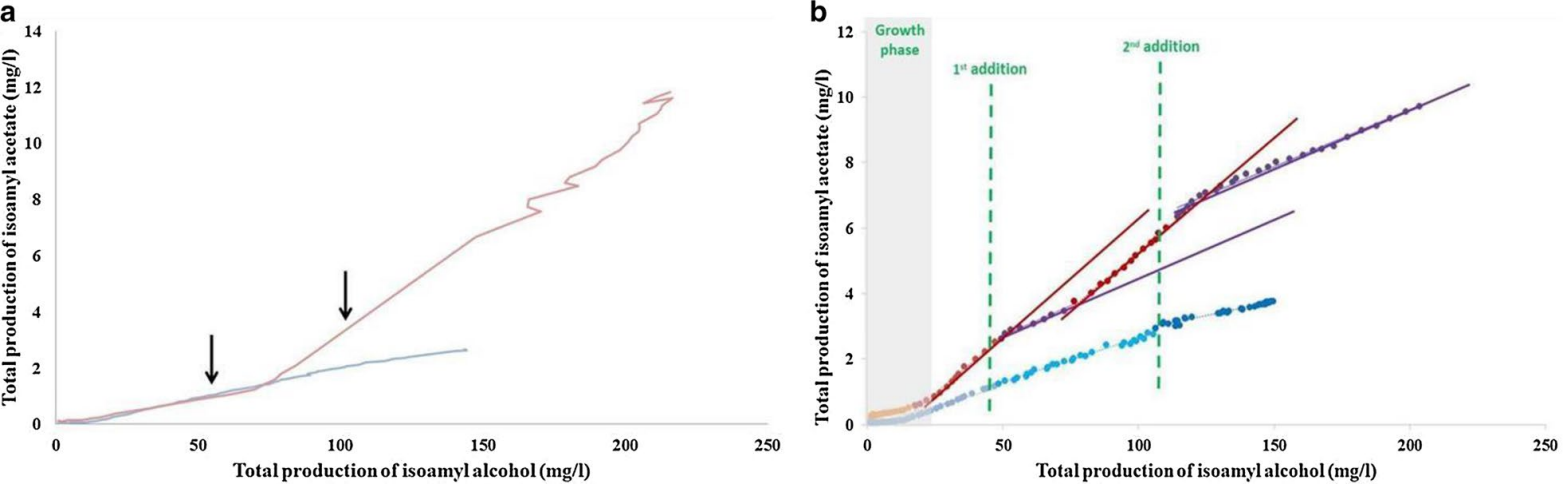
Focus on the bioconversion of isoamyl alcohol to isoamyl acetate. **a** Changes in total isoamyl acetate production as a function of total isoamyl alcohol production in SM330 with 8 mg/L of phytosterols for Lalvin EC1118^®^ (*blue*) and Affinity™ ECA5 (*red*). The *arrows* indicate the timing of sampling for transcriptomic analyses. **b** Changes in total isoamyl acetate production as a function of total isoamyl alcohol production subsequently to phytosterol additions for Lalvin EC1118^®^ (*blue*) and Affinity™ ECA5 (*purple and red*). For Lalvin EC1118^®^, despite phytosterol additions, bioconversion yield between the two compounds remains identical: 0.0241 (R^2^ = 0.947). For Affinity™ ECA5, four linear phases are identified; their yields of conversion are 0.0689 (R^2^ = 0.990); 0.0367 (R^2^ = 0.966); 0.0696 (R^2^ = 0.991); and 0.0351 (R^2^ = 0.955)

We studied two higher alcohol/acetate ester couples: isobutanol and isobutyl acetate; and isoamyl alcohol and isoamyl acetate. For these two couples and for both strains, the conversion yield was dependent on the initial nitrogen and phytosterol content: The highest yields were obtained at high nitrogen content (330 mg/l) and low phytosterol concentration (2 mg/l) (Table 2, Fig. 5a). For Lalvin EC1118^®^, the yield throughout the fermentation process was constant. By contrast, for Affinity™ ECA5, there were generally two production phases, with a yield comparable to that observed for Lalvin EC1118^®^ during the first phase and a much higher yield in the second phase (Table 2). The transition occurred during the stationary phase and was particularly visible with SM330 and 8 mg/l of phytosterols for Affinity™ ECA5 (Fig. 5a). One possible explanation for this drastic change in enzymatic activity is the presence of lipids that are known to repress the expression of *ATF1* [34].

**Table 2:** Production yields of acetate ester from its higher alcohol precursor

	SM70 2 mg/l	SM70 8 mg/l	SM330 2 mg/l	SM330 8 mg/l
*Production yield from higher alcohol (mg/mg) for Lalvin EC1118* ^*®*^				
Isobutyl acetate				
First phase	2.70 × l0^−3^	2.80 × l0^−3^	2.55 × l0^−2^	1.17 × l0^−2^
Second phase				
Isoamyl acetate				
First phase	1.90 × l0^−3^	1.60 × l0^−3^	4.40 × l0^−2^	2.04 × l0^−2^
Second phase	3.40 × l0^−3^	3.10 × 10^3^		
*Production yield from higher alcohol (mg/mg) for Affinity™ ECA5*				
Isobutyl acetate				
First phase	1.30 × l0^−3^	8.00 × 10^−4^	1.95 × l0^−2^	5.20 × l0^−3^
Second phase	1.02 × l0^−2^	8.90 × l0^−3^	6.51 × l0^−2^	4.21 × l0^−2^
Isoamyl acetate				
First phase	6.00 × l0^−3^	3.70 × l0^−3^	9.84 × l0^−2^	1.58 × l0^−2^
Second phase	1.75 × l0^−2^	1.15 × l0^−2^		7.07 × l0^−2^

To evaluate this hypothesis, we added phytosterol (8 mg/L) at different stages of the fermentation. These additions had no effect on the bioconversion of isoamyl alcohol to isoamyl acetate (Fig. 5b) and isobutanol/isobutyl acetate (data not shown) by Lalvin EC1118^®^. The corresponding bioconversion yields remained constant despite the additions. By contrast, the addition of phytosterols dramatically lowered the bioconversion of higher alcohols for Affinity™ ECA5. Indeed, after each lipid addition, the yield was divided by two (Fig. 5b).

**Fig. 6:**
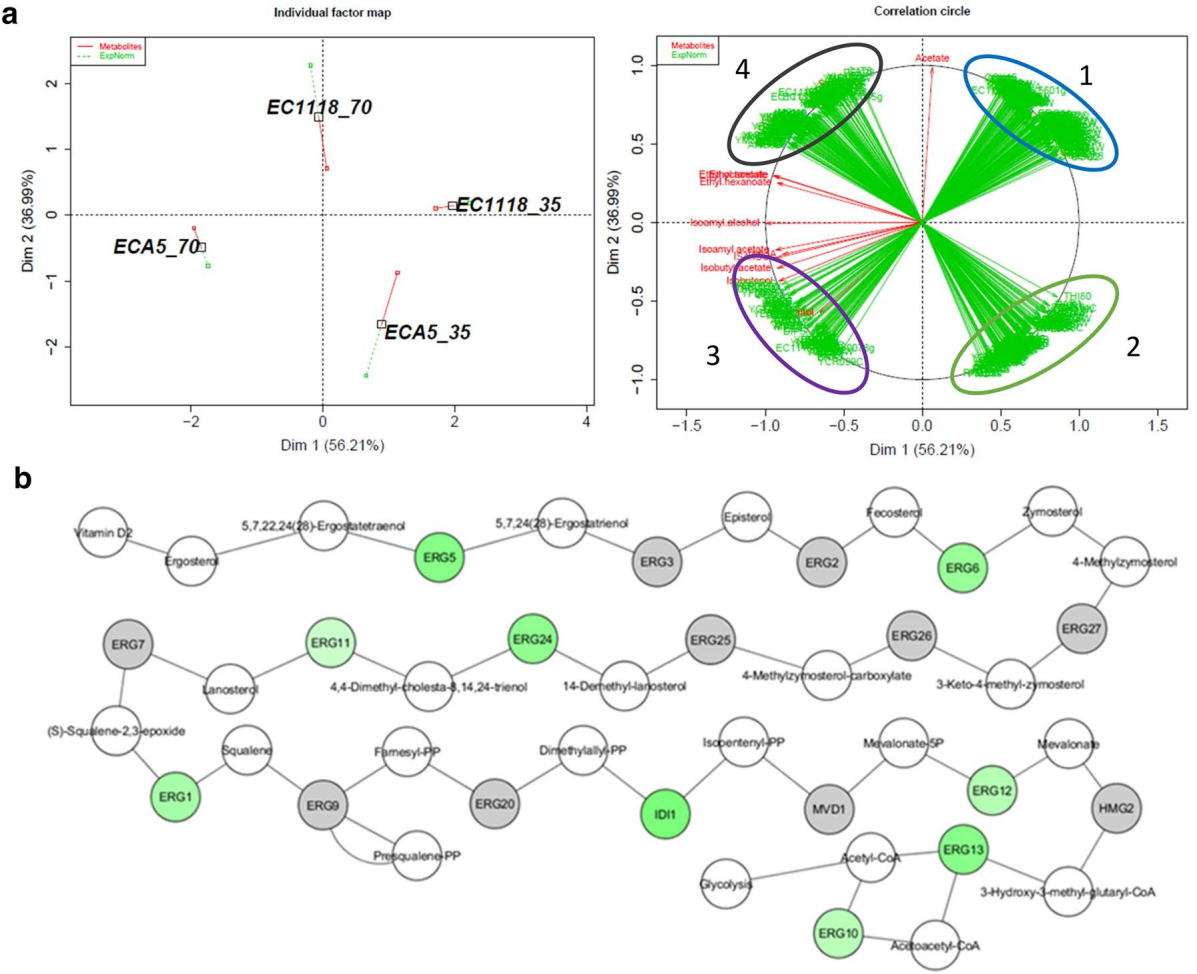
Modification of gene expression before (35 g/l of CO_2_ released) and after (70 g/l of CO_2_ released) the change in bioconversion yield between higher alcohols and acetate esters for the evolved strain**. a** Multivariate factorial analysis (MFA) of the genes obtained by sparse PLS-DA and 13 metabolites or ratios. Each fermentation is identified by the labels X and Y, where X corresponds to the strain and Y is the timing of sampling. *1*: Genes involved in sterol biosynthesis and oxido-reduction process; *2*: Genes involved in translation; *3*: Genes involved in cellular amino acid biosynthetic process, *4*: Genes involved in DNA repair. *ISA.ISO* ratio of isobutyl acetate to isobutanol, *IAA.IA* ratio of isoamyl acetate to isoamyl alcohol. **b** Comparison of expression of genes involved in the sterol biosynthesis pathway in Affinity™ ECA5 between the two sampling times (made by Cytoscape, [60]). *White circles* represent metabolites. *Grey circles* represent genes that are not differentially regulated. *Green circles* represent down-regulated genes

To better understand the underlying mechanisms, we performed a transcriptomic analysis of the two strains. Cells were sampled at 35 and 70 g/L of CO_2_ released (arrows in Fig. 5a). For Affinity™ ECA5, the first sample was collected before the change in bioconversion yield of isoamyl alcohol to its acetate ester and the second after this shift. A sparse PLS-DA analysis was performed with the normalized transcriptomic data and the production of all fermentative aromas. This approach first allowed for the selection of 500 genes (250 for each axis) that displayed the largest differences in their expression according to the strain or the sampling time. In the MFA representing the analysis (Fig. 6a), the first two dimensions accounted for 93 % of the total variation and clearly discriminated sampling times (among the first axis) and strains (among the second axis). The correlation circle highlighted four groups of genes with highly correlated expression (Fig. 6a, Additional file 2). A large portion of genes belonging to the first group were identified as involved in sterol biosynthesis and oxidation–reduction processes using the Genecodis3 program (Additional file 3). This group appeared to be negatively correlated with the third one that was enriched with genes related to cellular amino acid biosynthetic processes. These two gene clusters were the main contributors to the differentiation between strains. Affinity™ ECA5 exhibited a high expression of genes of group 3 combined with a down-regulation of those of group 1, unlike Lalvin EC1118^®^. The other groups, 2 and 4, predominantly consisted of genes related to translation and DNA repair, respectively. Genes of the second group were overexpressed at 35 g/L of CO_2_ released, whereas genes of the fourth one were down-regulated, regardless of the strain. These two latter groups allowed for the distinction of the sampling time. Interestingly, the sparse PLS-DA analysis showed a negative correlation between the genes involved in the sterol biosynthesis pathway and the aroma compounds: The expression of these genes was down-regulated when the concentration of fermentative aroma was maximal. In contrast, a positive correlation was found between the expression of these genes and the formation of acetate.

We then focused on differences between strains regarding the samparameter Weibull model is writtenpling times that corresponded to two different phases of acetate esters formation by Affinity™ ECA5, whereas the formation yield of these volatile molecules by Lalvin EC1118^®^ remained constant. To this end, we looked for variations between strains in pair-wise comparisons (with a fold change greater than 1.8) of the genes expression profiles obtained at the two sampling times (Additional file 4). Unexpectedly, no changes were observed in the expression of *ATF1* or *ATF2* in the evolved strain that could explain the increased formation of acetate esters at 70 g/L of CO_2_ released. In contrast, substantial differences were found in the expression of many genes involved in lipid metabolism, particularly in sterols biosynthesis, that were down-regulated in Affinity™ ECA5 (Fig. 6b). At the same time, several genes involved in glycogen and trehalose biosynthesis and transmembrane transport were overexpressed in the evolved strain.

## Discussion

The aim of our study was to better understand the metabolic reshaping of Affinity™ ECA5 caused by adaptive evolution [15] to compare the response of the evolved strain and its ancestral strain Lalvin EC1118^®^ to the modification of two key environmental parameters (nitrogen and phytosterols), combining on-line monitoring of aroma production and transcriptomic analysis.

An interaction between nitrogen and phytosterol content for both strains was observed; it resulted in differences in the kinetics profiles of the consumption of amino acids and the synthesis of fermentative aromas. The nitrogen/phytosterol interaction was stronger in SM330 than in SM70; this difference could be explained by the availability of lipids per cell. Indeed, in SM70, the population was lower; thus, each cell had enough lipids available. Conversely, in SM330, the yeast population was more important; the cells had fewer phytosterols available, making this parameter more discriminating. Moreover, this interaction was stronger for the strain Affinity™ ECA5 regardless of the concentration of nitrogen. An analysis of biomass composition showed that the lipid content was 2.5 times higher in the evolved strain (4.47 % g/g of dry weight) than in the ancestral strain (1.73 % g/g of dry weight) [15], suggesting a modification of lipid metabolism during the adaptive process.

Interestingly, the fermentation conditions had a different effect depending on the consumption of amino acids and on the class of studied compounds (higher alcohols, acetate or ethyl esters).

First, we could highlight differences on nitrogen assimilation rates depending on phytosterol content of the must and the yeast strain used (Fig. 2). The nitrogen sources for which the effect of phytosterols was different depending on the strain were mainly late consumed amino acids (according to the classification of Crépin et al. [31]) and ammonium. The carriers of these nitrogen sources are encoded by genes controlled by nitrogen catabolite repression (NCR). These observations suggest differences in the regulation of the consumption of these nitrogen sources between the evolved and ancestral strains. In line with these results, the comparison of the gene expression profiles of the two strains indicated an overexpression of the genes *MEP2* (coding for an ammonium transporter) and *GAP1* (coding for the carrier of alanine, arginine and glycine) in the evolved strain. The adaptive evolution could have therefore triggered changes in the transcriptional or post-transcriptional regulation of the NCR-regulated carriers.

The phytosterol content of the must also affected the consumption efficiency of nitrogen sources by the two strains. This effect could be explained by changes in the plasma membrane due to the incorporation of phytosterols. A significant proportion of these sterols in the membrane could perturb its properties and disrupt its structure [35–37]. Moreover, previous studies have shown that lipids (especially ergosterol and sphingolipids) can form micro-domains in the membrane called lipid rafts that are important for protein sorting [38–40]. Several nutrient transporters have been located in these domains in *S. cerevisiae*, particularly the arginine permease Can1p and the general amino acid permease Gap1p [41, 42]. Phytosterols with a structure similar to that of ergosterol could play a similar role, thus explaining the differences in consumption of amino acids depending on the lipid dose in the medium.

Previous studies have demonstrated that the characteristic property of the Affinity™ ECA5 is a marked increase in fermentative aroma formation [15, 18, 19]. Herein, we show that these traits are generally preserved under various conditions. The final formation of volatile compounds by the evolved strain is substantially higher compared to that observed for Lalvin EC1118^®^, except for ethyl esters, which are produced at the same level by the two strains when nitrogen is limiting.

The differentiation of the strains on the basis of ethyl esters was only visible in SM330. These findings are in line with the fact that ethyl esters derived from lipid metabolism. Indeed, in SM70, the amount of biomass formed is less important, and the lipid requirement lower. Therefore, exogenous lipids are sufficient to meet this requirement, even at 2 mg/L, and the de novo synthesis of lipids is low. Conversely, in SM330, the lipid requirement for biomass formation is higher and thus, the de novo synthesis provides a greater contribution. Affinity™ ECA5 produced 2.5 times as much lipid as Lalvin EC1118^®^ [15], indicating a modification of its lipid metabolism. This likely means a difference in the regulation of lipid metabolism that might explain the overproduction of ethyl esters in a nitrogen-rich environment.

The response of higher alcohols was complex and dependent on the studied compounds. Propanol production, quite similar between the two strains [19], was proportional to the initial nitrogen content as observed in previous studies [4, 29, 43, 44]. For isobutanol and isoamyl alcohol, systematic overproduction by Affinity™ ECA5 was observed. The overproduction of higher alcohols by the evolved strain can be explained by stronger activation of the biosynthesis pathways of amino acid precursors, consistent with Affinity™ ECA5′s overexpression of genes involved in amino acids biosynthetic processes (*ARG1*, *ARG3*, *ARG7*, *ARG8*, *CPA2*) identified by transcriptomic analysis. The pool of ketoacids could be more important in this strain and would be directed toward the synthesis of higher alcohols. This hypothesis is in line with the results plotted by MFA (Additional file 1): the relation between amino acid exhaustion and the maximal production rate of volatile molecules varies considerably between the two strains.

Concerning acetate esters, systematic overproduction was observed for Affinity™ ECA5. The changes in environmental conditions led to a similar response for both strains. The maximal production and the maximal specific rate of acetate esters were reached in the nitrogen-rich medium at low phytosterol content, consistent with the literature [4, 29, 34, 45–47]. The study of specific rates confirmed the effect of the strain and of the environmental parameters on the productions of these molecules. At high nitrogen content, the profiles of the specific rates of acetate ester production were governed by the lipid dose, showing the predominance of the environmental effects. The factors that affected the flux of ester synthesis were the same for both strains. Conversely, in SM70, the strain effect was dominant.

We studied the bioconversion of higher alcohols to their acetate esters more precisely. Overall, throughout the entire fermentation process, this bioconversion was greater for Affinity™ ECA5 than for Lalvin EC1118^®^. The evolved strain presented two consecutive yields, with the highest value for the second one, whereas the parental strain showed a constant yield during the fermentation. Several hypotheses may explain this enhanced conversion. The enhanced conversion can be attributed to a greater availability of precursors—higher alcohol and/or acetyl-CoA—and/or to increased enzymatic activity of acetyltransferases.

Several results obtained in this study are consistent with an effect related to the modification of lipid metabolism, particularly the effect on the availability of acetyl-CoA. First, for Affinity™ ECA5, 8 h after the first addition of phytosterols, the bioconversion yield returned to its maximal value, suggesting consumption of these sterols and thus modified management of phytosterols by the evolved strain. Second, the comparison of gene expression profiles of Affinity™ ECA5 between the two sampling times did not reveal modification of the expression of the *ATF1* and *ATF2* genes encoding the acetyltransferases. In contrast, down-regulation of genes involved in the sterol biosynthesis pathway was observed after the change in bioconversion yield in the evolved strain. Finally, differences in the expression of various genes involved in the synthesis of acetyl-CoA (*ALD4*, *ALD6* and *ACS1*, *ACS2*) were found between the two strains (Additional file 5).

Based on all these data, we propose the following scenario. Affinity™ ECA5 more efficiently assimilates phytosterols present in the medium. As a consequence, acetyl-CoA is less utilized to produce sterols and is more available to react with higher alcohols to produce more acetate esters. Nevertheless, the increase in availability in acetyl-CoA did not necessarily result in an increase in ethyl ester production because acetyl-CoA was not used as a direct substrate for the synthesis of these compounds, as is the case for acetate ester synthesis. Indeed, to produce ethyl esters from acetyl-CoA, certain steps were required: (1) elongation into acyl-CoA followed by (2) an esterification reaction with ethanol. Moreover, acyl-CoA can be converted to fatty acids incorporated into the biomass. For this last reason, a larger pool of acyl-CoA did not necessarily result in a higher production of ethyl esters; but in a greater accumulation of lipids.

The hypothesis related to a higher availability of acetyl-CoA is consistent with the known Km for the enzyme Atf1p: 29.8 mM for isoamyl alcohol and 0.025 mM acetyl-CoA [48]. A change in the pool of acetyl-CoA, even a minor one, could therefore have a major effect on the conversion of higher alcohol acetate esters. This hypothesis is supported by results recently obtained by Bloem et al. [49] showing the effect of the availability of acetyl CoA on the synthesis of esters after a modification of the redox status of the cell.

## Conclusion

In this study, we combined gene expression analysis with a dynamic study of the synthesis of fermentative aromas to compare the performances of the evolved strain Affinity™ ECA5 and its ancestral strain Lalvin EC1118^®^. This study revealed differences between the two strains at different levels. We highlighted certain differences in nitrogen source consumption suggesting modifications of the NCR in the evolved strain. These changes appeared to be related to adaptive evolution. Indeed, Crépin et al. [31] highlighted that the sequence of amino acid assimilation is highly conserved in *S. cerevisiae* species. We also observed differences in the dynamics of fermentative aroma production, especially for higher alcohols. These kinetic differences suggested that the intracellular pool of keto acids was more important in the evolved strain and redirected more heavily towards the synthesis of higher alcohols. The study of the bioconversion of higher alcohols to acetate esters revealed marked differences between the evolved strain and its ancestral strain. Through a combined analysis of dynamic and transcriptomic data, a variation in the manner in which the lipid source was managed by the evolved strain was underlined. This metabolic modification was particularly visible in the bioconversion of higher alcohols to acetate esters and might be caused by differences in the availability of acetyl CoA. This result is consistent with the increased flux from acetate towards acetyl-CoA and lipid synthesis in Affinity™ ECA5 [15].

## Methods

### Yeast strains

The *S. cerevisiae* yeast strains used in this study are the commercial strains Lalvin EC1118^®^ and Affinity™ ECA5 (Lallemand SA, Montreal, Canada), obtained by adaptive evolution of Lalvin EC1118^®^. Fermentation flasks were inoculated with 10 g/hl active dry yeast previously rehydrated for 30 min at 37 °C in a 50 g/L glucose solution (1 g of dry yeast diluted in 10 ml of this solution).

### Fermentation media

Fermentation was carried out in synthetic medium (SM) that simulates standard grape juice [50]. The SM used in this study contained 200 g/l of sugar (100 g/L of glucose and 100 g/L of fructose); 6 g/L of malic acid; 6 g/L of citric acid; 750 mg/L of KH_2_PO_4_; 500 mg/L of K_2_SO_4_; 250 mg/L of MgSO_4_.7H_2_O; 155 mg/L of CaCl_2_.2H2O; 200 mg/L of NaCl; vitamins (mg/L): myo-inositol (20), calcium pantothenate (1.5), thiamin hydrochloride (0.223), nicotinic acid (2), pyridoxine (0.25), and biotin (0.003); and oligoelements (mg/L): MnSO_4_.H_2_O (4), ZnSO_4_.7H_2_O (4), CuSO_4_.5H_2_O (1), CoCl_2_.6H_2_O (0.4), H_3_BO_3_ (1), and (NH_4_)_6_Mo_7_O_24_ (1). The pH of the medium was adjusted to 3.3 with NaOH 10 M.

The nitrogen source was composed of ammonium chloride and amino acids. We used three concentrations of assimilable nitrogen: 70, 200 and 330 mg/L. The composition of the stock solution of amino acids was as follows (in g/L): tyrosine (1.4), tryptophan (13.7), isoleucine (2.5), aspartate (3.4), glutamate (9.2), arginine (28.6), leucine (3.7), threonine (5.8), glycine (1.4), glutamine (38.6), alanine (11.1), valine (3.4), methionine (2.4), phenylalanine (2.9), serine (6.0), histidine (2.5), lysine (1.3), cysteine (1.0) and proline (46.8). To obtain 70 mg/l of assimilable nitrogen in the MS, 2.16 ml of this solution and 75 mg/L of NH_4_Cl were added to the medium; for 200 mg/L, 6.16 ml of amino acid solution and 220 mg/L of NH_4_Cl were added, and for 330 mg/L, 10.16 ml of amino acid solution and 360 mg/L of NH_4_Cl were added.

The SM medium was initially supplemented with three different concentrations of phytosterols (85,451, Sigma Aldrich): 2, 5 and 8 mg/L to satisfy the lipid requirements of yeast cells during anaerobic growth. The stock solution was composed of 15 g/L of phytosterols in Tween 80 and ethanol (1:1, v/v). Phytosterol additions during fermentation were performed at 8 mg/L.

### Fermentation conditions

Fermentations were performed in 300 ml fermenters with musts containing three levels of assimilable nitrogen (70, 200 and 330 mgN/L) and three levels of phytosterols (2, 5 and 8 mg/L) at three temperatures (20, 24 and 28 °C). The data presented in the box plot (Fig. 1) were obtained in a previous study on Lalvin EC1118^®^ [29]. The same Box-Behnken design [29] was used to study the response of Affinity™ ECA5 to changes in fermentation conditions. The concentrations of volatile compounds in the liquid phase were measured by GC–MS using the method described in [29].

Fermentations were run in 10 L stainless steel tanks at 24 °C. The amount of CO_2_ released was measured accurately and automatically with a gas mass flow meter to calculate the rate of CO_2_ production (dCO_2_/dt). Anaerobiosis was obtained by bubbling argon into the medium.

Each fermentation was performed once, except for the condition involving 330 mg/L of nitrogen and 8 mg/L of phytosterols, for which fermentation was performed in duplicate. We previously determined that experiments run with this online monitoring system yield highly reproducible results [4, 51]. In this work, for the various volatile compounds assessed, the relative standard deviation (SD) between duplicates was very low throughout the fermentation process: 3 % for propanol, 4 % for isobutanol, 4 % for isoamyl alcohol, 2 % for ethyl acetate, 3 % for isobutyl acetate, 5 % for isoamyl acetate, 4 % of for ethyl hexanoate and 5 % for ethyl octanoate.

### Cell population

During fermentation, the cell population was determined using a Coulter counter (Model Z2, Beckman-Coulter, Margency, France) fitted with a 100 μm aperture probe.

### Measurement of assimilable nitrogen

The ammonium concentration was determined enzymatically (R-Biopharm, Darmstadt, Germany).

The free amino acid content of the must was determined by cation exchange chromatography, with post-column ninhydrin derivatization (Biochrom 30, Biochrom, Cambridge, UK) as described by Crépin et al. [31].

### Analysis of volatile compounds

The concentrations of volatile compounds in the headspace of the tank were measured with an online GC device. Headspace gas was pumped from the tank at a flow rate of 14 ml/min, through a heated transfer line. Carbon compounds were concentrated in a cold trap (Tenax TM) for 6 min, desorbed at 160 °C for 1 min, and analyzed with a Perichrom PR2100 GC coupled to a flame ionization detector (Alpha MOS, Toulouse, France). The details of the GC method and the calibration procedure were as previously described by [4, 33].

### Volatile compound balances during fermentation

#### Concentrations in the liquid

The concentration of a volatile compound in the liquid [*C*^*liq*^(*t*)] was calculated from the concentration measured online in the gas phase, expressed as *C*^*gas*^(*t*) in mg/l CO_2_, using the partition coefficient (*k*_*i*_) value (Eq. 1):

1 $$C^{liq} \left( t \right) = \frac{{C^{gas} \left( t \right)}}{{k_{i} }}$$

The value of *k*_*i*_ (Eq. 2) was calculated using the model developed by [30] as a function of the fermenting must composition, characterized by ethanol concentration and temperature:

2 $$lnk_{i} = F1 + F2 \times E - \frac{F3 + F4 \times E}{R}\left( {\frac{1000}{T} - \frac{1000}{{T_{ref} }}} \right)$$

where *E* is the ethanol concentration (g/L) in the liquid phase, calculated from the measurement of the amount of CO_2_ released, which is proportional to sugar consumption; *T* is the current absolute temperature; and *T*_*ref*_ is the absolute reference temperature (i.e., 293.15 K, or 20 °C in this study). *F1, F2, F3* and *F4* are constants identified for each volatile compound. The values of these parameters for the various molecules considered were determined by Mouret et al. [4, 33].

#### Losses in the exhaust gas

Losses into the exhaust gas were calculated using Eq. 3:

3 $$L\left( t \right) = \int_{0}^{t} {C^{gas} \left( t \right) \times Q\left( t \right) \times dt}$$

where *Q*(*t*) is the CO_2_ flow rate at time *t*, expressed in l CO_2_/l must/h.

The relative loss (RL), expressed as a percentage of total production (*P* (*t*)), is determined as follows (Eq. 4):

4 $$RL = \frac{L\left( t \right)}{P\left( t \right)} = \frac{{\int_{0}^{{t_{end} }} {C^{gas} \left( t \right) \times Q\left( t \right) \times dt} }}{{C^{liq} \left( {t_{end} } \right) + \int_{0}^{{t_{end} }} {C^{gas} \left( t \right) \times Q\left( t \right) \times dt} }}$$

where *t*_*end*_ is the final fermentation time in hours.

#### Total production

The total production of a volatile compound at time *t*, expressed as *P*(*t*) in mg/L must, was calculated by adding the concentration in the liquid phase, expressed as *C*^*liq*^(*t*) in mg/L must, to the amount of the volatile compound lost in the gas phase, expressed as *L*(*t*) in mg/L must (Eq. 5):

5 $$P\left( t \right) = C^{liq} \left( t \right) + L\left( t \right)$$

This production value represents the capability of the yeast to produce a volatile compound, independently of the subsequent fate of the compound—accumulation in the liquid phase or evaporation.

### Data processing and statistical analyses

Statistical analysis was performed with R software, version 3.1.1 [52].

We obtained three datasets, in which each variable of interest is a curve along the time (h) that we expressed in terms of consumed sugar (g/L). We chose to summarize these three datasets by modeling each curve with an adequate model and then extracting criteria of interest.

First, for each condition, the biomass was modeled using a Weibull model with the drc package [53]. The one four-parameter Weibull model is written as follows:

6 $$f\left( x \right) = c + \left( {d-c} \right)\left[ {1-exp^{{ - exp^{{\left[ {b\left( {\ln \left( x \right) - \ln \left( e \right)} \right)} \right]}} }} } \right]$$

This four-parameter ascending function is asymmetric with an inflection point at time e. For each modeled function, we extracted several criteria of interest: µmax, defined as the maximal of the ratio f’(t)/f(t) for each t, expressed in h^−1^; the inflection point, expressed in terms of consumed sugar g/L; and the maximum biomass, expressed in 10^6^ cells.

Considering amino acid (AA) consumption, we modeled each AA under each condition with the drc package and a Weibull model. The four-parameter Weibull function is written as follows:

7 $$f\left( x \right) = c + \left( {d-c} \right)exp^{{-exp^{{\left[ {b\left( {\ln \left( x \right) -\ln \left( e \right)} \right)} \right]}} }}$$

This four-parameter decreasing function is asymmetric with an inflection point at time e. For each modeled function, we extracted the following criteria: the maximal rate, which is the maximum of the first derivative of the function expressed in mg/L.h, and the inflection point and the point at which the quantity of AA is null (called Point.AA0), both expressed in terms of consumed sugar (g/L).

For these two parametric models, the normality of residual distributions and homogeneity of variance were studied with standard diagnostic graphs; no violation of the assumptions was detected.

Each volatile compound under each condition was then modeled using a non-parametric model using the cellGrowth package [54]. The model used is a local regression and allows for the extraction of the inflection point expressed in consumed sugar (g/L), the maximal production in mg/L and the maximal rate (maximum of the first derivative in mg/L.h). To calculate the specific rate, we divided the first derivative of the model (the rate) by the population, as estimated above. Finally, we recorded the maximum specific rate (SRmax) and the time at which this maximum was reached, expressed in consumed sugar (g/L) (PointSRmax).

To provide an overview of the dataset, principal component analysis (PCA) was carried out with the FactoMineR package [56].

Multivariate factorial analysis (MFA) was then performed for the two strains (Lalvin EC1118^®^ and Affinity™ ECA5) at two levels of nitrogen (70 and 330 g/L) and two levels of phytosterols (2 and 8 mg/L). This analysis allowed for the study of links between the consumption of AA and volatile compound production [55].

### Gene expression analysis

For each fermentation condition (SM330, 8 mg/L of phytosterols with the two strains), three independent fermentations were carried out in parallel and sampled when CO_2_ production reached 35 and 70 g/L, corresponding to two different phases of aroma metabolism. Cells (1x10^9^ cells) were harvested by centrifugation at 1000*g* for 5 min at 4 °C, and the cell pellets were washed with DEPC-treated water and then frozen in methanol at -80 °C. Total RNA was extracted with Trizol reagent (Gibco BRL, Life Technologies) and was purified with the RNeasy kit (Qiagen). The quantity and quality of the extracted RNA were verified by spectrometry (NanoDrop 1000, Thermo Scientific). We used the Agilent 8 × 15 k gene expression microarrays (Design ID 038619 with 40 EC1118-specific genes, Agilent Technologies, Santa Clara, CA, USA) according to the manufacturer’s instructions. Fluorescent cRNAs were synthesized from 100 ng of total RNA using the One color RNA Spike-In kit (Agilent Technologies). Labeled cRNA was purified with the RNeasy Kit (Qiagen). Microarrays were hybridized for 17 h at 65 °C in a rotating hybridization oven (Corning) with the Gene Expression Hybridization kit (Agilent). The hybridization signal was detected with a GenePix 4000B laser Scanner (Axon Instruments).

The limma package [56] was used to import and normalize the global microarray data (quantile method for normalization between arrays). The entire dataset is available in the “Gene Expression Omnibus Database” (No. GSE68354). Transcriptomic data were analyzed by two different methods.

For each level of CO_2_ released (35 and 70 g/L) and based on this normalized dataset of 6200 expression data for the two strains, we used sparse partial least squares—discriminant analysis (sPLS-DA), which is an exploratory approach in a supervised context, to select the most important transcripts relative to the four samples [57]. We tuned the number of dimensions of the sPLS-DA to two and the number of variables to choose on these two dimensions to 500 (250 for each).

Functional analysis was performed on the selected transcripts by time point to highlight significant functional groups according to the gene ontology (GO) process terms using the Genecodis program [58] via the FDR method at a p value cutoff of 0.05 [59].

For each time point, MFA was then performed to obtain an overview of the dataset, which consisted of 513 variables measured for the two strains (Lalvin EC1118^®^ and Affinity™ ECA5) and for the two sampling times. The dataset included a set of individuals described by two types of variables: the normalized expression of the 500 transcripts selected by the sPLA-DA according to the two strains and the 13 compounds (or ratios) produced during fermentation by the two strains. The MFA took the structure of the two groups of data into account and balanced the effect of each group of variables, enabling the study of links between expression data and volatile compounds production [55].

To determinate the differential gene expression between experimental conditions, a modified t-test was performed by filtering on confidence at p < 0.05, using the Benjamini and Hochberg false discovery rate as multiple testing corrections of the t-test p values [59]. The genes with different levels of expression were grouped according to gene ontology (GO) process terms using the Genecodis program [58].

## Additional files

10.1186/s12934-016-0434-6 Multivariate factorial analysis (MFA) with the timing when amino acid was exhausted (Point.AA0) and the timing corresponding to the maximal specific rate of total production of aromas (PointSRmax). Each fermentation is identified as X, Y, Z, where X corresponds to the strain, Y to the initial nitrogen concentration in mg N/l and Z is the phytosterol content in mg/l. PR: propanol; ISO: isobutanol; IA: isoamyl alcohol; EA: ethyl acetate; ISO: isobutyl acetate; IAA: isoamyl acetate; EH: ethyl hexanoate; EO: ethyl octanoate; Leu: leucine; Val: valine; Thr: threonine; NH4: ammonium; Nass: initial assimilable nitrogen.
10.1186/s12934-016-0434-6 List of genes composing each group obtained by sparse PLS-DA.
10.1186/s12934-016-0434-6 Genecodis classification of genes selected by sparse PLS-DA into biological processes. NG: number of genes in our list; NGR: total number of genes in a category.
10.1186/s12934-016-0434-6 Genes differentially expressed between the two sampling times for Affinity™ ECA5.
10.1186/s12934-016-0434-6 Genes differentially expressed between Affinity™ ECA5 and Lalvin EC1118^®^ for the first time of sampling.
