# Supplementary figures and images for: Key role of lipid management in nitrogen and aroma metabolism in an evolved wine yeast strain
Source: Microb Cell Fact. 2016 Feb 9;15:32. doi: 10.1186/s12934-016-0434-6 (PMC4748530; doi:10.1186/s12934-016-0434-6)

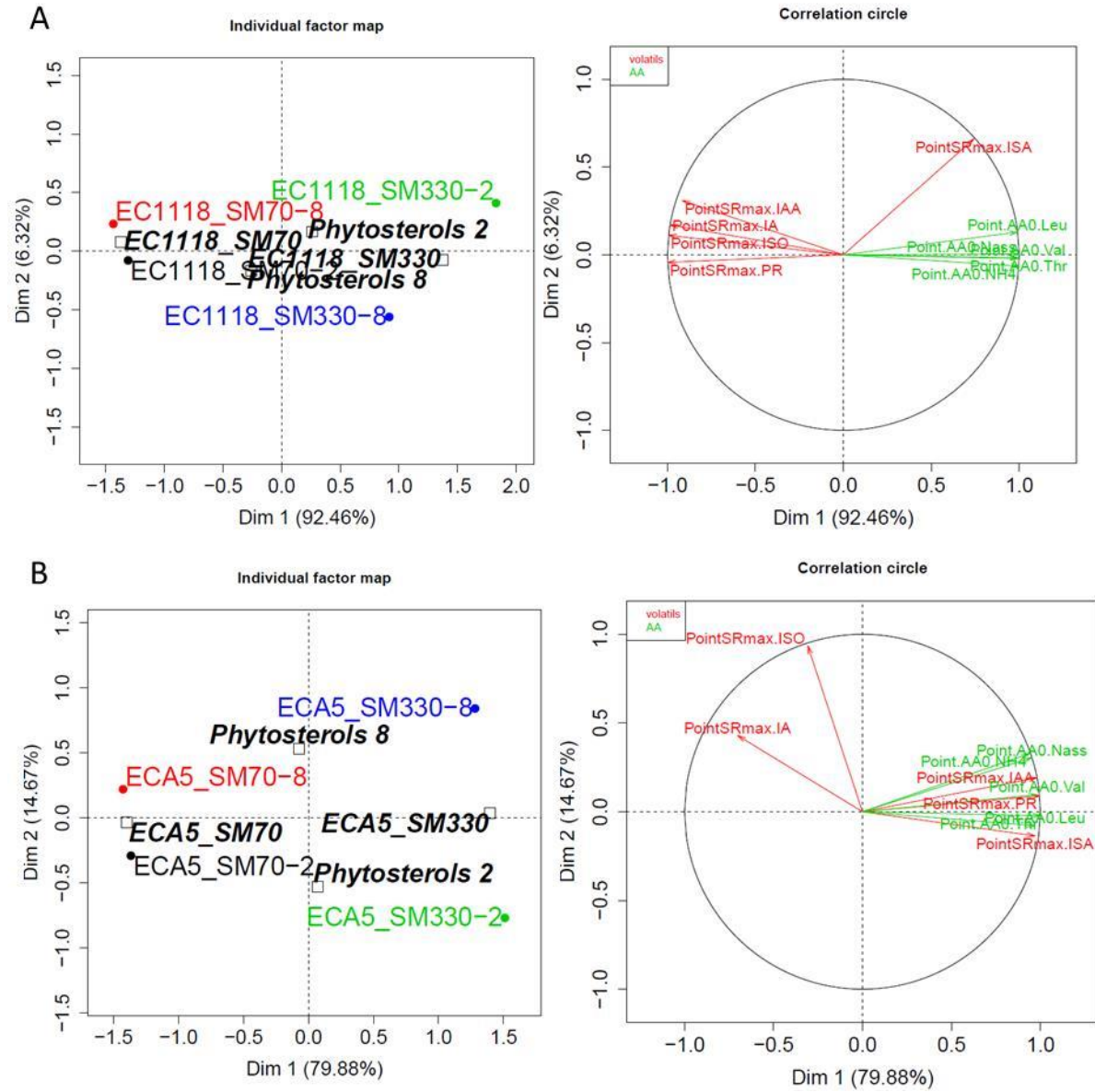

Supplement: Supplementary file 1 — 10.1186/s12934-016-0434-6 Multivariate factorial analysis (MFA) with the timing when amino acid was exhausted (Point.AA0) and the timing corresponding to the maximal specific rate of total production of aromas (PointSRmax). Each fermentation is identified as X, Y, Z, where X corresponds to the strain, Y to the initial nitrogen concentration in mg N/l and Z is the phytosterol content in mg/l. PR: propanol; ISO: isobutanol; IA: isoamyl alcohol; EA: ethyl acetate; ISO: isobutyl acetate; IAA: isoamyl acetate; EH: ethyl hexanoate; EO: ethyl octanoate; Leu: leucine; Val: valine; Thr: threonine; NH4: ammonium; Nass: initial assimilable nitrogen. [file 12934_2016_434_MOESM1_ESM.pdf]
