# Supplementary material for: Key role of lipid management in nitrogen and aroma metabolism in an evolved wine yeast strain
Source: Microb Cell Fact. 2016 Feb 9;15:32. doi: 10.1186/s12934-016-0434-6 (PMC4748530; doi:10.1186/s12934-016-0434-6)
Supplement: Supplementary file 2 — 10.1186/s12934-016-0434-6 List of genes composing each group obtained by sparse PLS-DA. [file 12934_2016_434_MOESM2_ESM.pdf]

Group 1	Group 2	Group 3	Group 4
COQ2	PHO11	YCR099C	ARG82
MDM38	GDT1	BNA3	YPT52
PEX21	SLD5	TIR2	MTC4
BUD21	RPL18A	ADE1	IRC20
GFD2	RPS1B	ALG7	YEL1
CNS1	RPL24A	YDR089W	INO1
BUD20	BIO4	ADE17	UBC8
FUI1	PHO12	IDP1	CRP1
SFK1	HIS7	CPA2	ATG20
YAP7	RPP2A	VHC1	YML116W.A
RRN7	RPL42A	EC1118_1F14_0078g	CWC24
YNL234W	ARD1	RNR3	YNL092W
RCL1	RPS29A	RSC9	FMP21
ETT1	PHA2	VMA6	VAC7
NCS2	YOL159C	MET12	YKL071W
HRB1	RPP1A	YER134C	YLR312C
URA3	RPL4A	ENT3	PCD1
HEL2	TPN1	YER135C	RTF1
CIR2	RPS10B	ARG8	ENO1
ATP14	VBA4	RAD59	PIB1
RNH203	NIT3	RNR4	HOC1
IMP3	YDL158C	YNR068C	CSR2
RCM1	BCH1	RPN10	YRF1.6
YMR166C	UBC12	DIP5	IWR1
MEF1	YHL044W	FIG1	LGE1
DRS1	YCR087C.A	SUL2	YRF1.8
YPL068C	RPL23B	YDR090C	LIP5
BAS1	RPS26B	HUL4	VHS1
RPC82	BUD16	EST3	SNA4
CYC8	THI11	STU2	RSF1
IDP2	BUD17	TUB3	MEK1
HIP1	RPS13	MPC2	SNT309
SAH1	RPS11A	SYN8	SKM1
GEP3	RPL19A	VPS51	RIM8
ZRT1	PRD1	ARG1	YPR204W
TRM1	ENV10	VPS17	GSC2
NTE1	RBS1	ADY3	NTG2
AAD4	APT1	GUD1	SPI1
SAR1	RPL12B	ADH5	BSD2
YOR378W	RPS16A	PPM1	YBL086C
IMP4	PMT6	PCM1	PIN3
UBP10	HEM3	VPS54	YDR186C
YGL188C	RPL26A	SEC2	MLH3
ARE2	RPS10A	CEM1	RMD5

FTR1	DPS1	YER184C	SPO14
MOT3	RNA1	ARG7	SAP155
RIB2	SNL1	VBA1	PUT4
MSC7	POL5	STV1	SHE10
ERG12	CCT4	IRS4	RTT102
HXT4	RPL6B	YGL082W	RAD4
PXR1	RPL35A	TOS4	CTH1
AFI1	RPL20B	LHS1	SPO22
DPL1	YGL039W	SRY1	JHD1
MRPL44	YNR066C	FUS1	URN1
BUR2	PUS7	ERJ5	YIG1
GOT1	NAT1	LSB5	UPS3
MRPL6	RPL7A	RMD6	DAS1
YBR242W	SIP3	TPD3	SLX8
AIM14	BIO3	ARP6	GAT2
ACS2	COG1	FES1	YKL050C
ADH6	GIM5	DDI1	YIL024C
CWC2	RPL43B	YFL065C	YKL133C
ROX1	RPL37B	ARG3	VPS72
ESF2	DPM1	YOR385W	BDP1
ATP16	FRS1	YAR023C	DON1
MET8	RPL11B	YLR257W	YPT53
ECM7	RPL31B	YGR190C	HSP30
PET309	YCR015C		RAD7
YOL029C	DML1		NDC80
NCA3	THI13		RPI1
SMI1	SEC12		CLF1
SCC2	BPL1		BAG7
ERG2	RPL33B		MSN4
NAF1	YHL042W		RSB1
YPL216W	SSH1		MND1
HSP12	RPS29B		YLR311C
MIC60	SXM1		AIM23
YBR096W	MED1		ABM1
ERG29	RPL4B		OSW1
ERG13	RPL9B		MPS2
KTI12	RPL11A		VPS24
HMG1	WTM2		LIN1
KTI11	LCB1		SKG1
ATP17	ALG6		SLM1
HFA1	VPS75		VHS3
OCA5	KIN3		DOT6
YDR222W	MYO5		EC1118_1O4_6667g
MRPL20	RPL24B		GAL2
MNN5	TRS65		RPN4
ATP25	RPL36A		EC1118_1N26_0045g
YDR056C	YML018C		TOS8

PSY2	SRP68	BYE1
ERG6	LOT5	PDC1
RMD8	TIM50	YIL077C
GND2	CTR9	KRE28
YLH47	GRS1	ATG1
AEP2	MMT1	YDR262W
CYC1	RPL27B	EC1118_1O4_6656g
HYP2	RVB2	GIP1
YDL086W	RPS28B	ZEO1
DBP2	PRT1	YTA7
ALP1	TIF3	MKK2
YOR062C	PRS1	PHO80
HPF1	SHR3	YML020W
YJL181W	YOR225W	GDH3
ERG20	RPL22A	POP6
COR1	HMF1	YGR122C.A
FET3	TRM732	AHC1
ERG11	RRN6	YMR295C
CBP1	RPS30A	NAB2
ANP1	YBR178W	RAD28
YHR078W	LAG1	YHL050C
ERG5	KAP104	YLR345W
OMA1	TAH18	TEN1
AAH1	RPS21A	MCH2
DRE2	TCD1	PHM8
AVO2	MET18	UFD1
CYB5	PML39	YDR306C
ERG26	YDR327W	IDP3
SFM1	YDR514C	SML1
ERT1	TRM10	MDM32
NOP53	RKR1	YLR271W
ERG1	GAR1	YNL194C
MDV1	THI80	YLL020C
PSR1		PEX28
ACN9		CAT8
MRPL39		
FAA1		
IZH1		
MTR4		
HXT3		
SLD3		
NDI1		
AQR1		
YLR224W		
MGM101		
SCM4		
RCF2		

QCR2
MLS1
CIN5
FCY2
MZM1
YLR407W
CYB2
CYC3
UBX6
RSM22
ASG1
SLP1
PET9
MRPL51
QCR7
RTC6
TSA1
NQM1
EC1118_1O4_6601g
IFH1
UPS2
IZH3
PKH1
ERG28
HPA2
YME2
COX8
YER053C.A
CTT1
FMP48
CCC2
DLD1
YGR079W
EST2
COX6
HFD1
YHR202W
DIA1
PDE2
CTR3
YTP1
STB5
COX4
RIP1
COX15
