# Supplementary material for: Key role of lipid management in nitrogen and aroma metabolism in an evolved wine yeast strain
Source: Microb Cell Fact. 2016 Feb 9;15:32. doi: 10.1186/s12934-016-0434-6 (PMC4748530; doi:10.1186/s12934-016-0434-6)
Supplement: Supplementary file 3 — 10.1186/s12934-016-0434-6 Genecodis classification of genes selected by sparse PLS-DA into biological processes. NG: number of genes in our list; NGR: total number of genes in a category. [file 12934_2016_434_MOESM3_ESM.pdf]

	Category	p-value	NG	NGR	Genes
Group 1	ergosterol biosynthetic process [GO:0006696]	4.81E-10	11	23	ERG11 ERG20 ERG28 ERG26 ERG6 ERG12 ERG5 ERG2 ERG1 ERG13 HMG1
	sterol biosynthetic process [GO:0016126]	2.13E-09	11	27	ERG11 ERG20 ERG28 ERG26 ERG6 ERG12 ERG5 ERG2 ERG13 HMG1 CYB5
	steroid biosynthetic process [GO:0006694]	1.57E-08	10	25	ERG11 ERG20 ERG28 ERG26 ERG6 ERG12 ERG5 ERG2 ERG13 HMG1
	lipid biosynthetic process [GO:0008610]	2.33E-07	12	52	ERG11 ERG20 ERG28 ERG26 ERG6 ERG12 ERG5 ERG2 ERG13 HMG1 HFA1 SFK1
	oxidation-reduction process [GO:0055114]	5.94E-06	24	284	ERG11 MET8 CIR2 CTT1 AIM14 ERG26 ADH6 IDP2 COX8 CYB2 ERG5 MTR4 HMG1 AAD4 FET3 HFD1 ERG1 GND2 NDI1 TSA1 MSC7 DLD1 COX15 RIP1
	aerobic respiration [GO:0009060]	7.30E-05	10	59	CBP1 QCR2 QCR7 MRPL51 COR1 COX4 PET309 PET9 DLD1 RIP1
	electron transport chain [GO:0022900]	1.27E-04	9	50	CYB5 QCR2 QCR7 CIR2 AIM14 CYB2 COR1 CYC1 RIP1
	isoprenoid biosynthetic process [GO:0008299]	2.30E-04	5	12	COQ2 ERG20 ERG12 HMG1 ERG13
	mitochondrial electron transport ubiquinol to cytochrome c [GO:0006122]	3.56E-03	4	11	QCR2 QCR7 CYC1 RIP1
	mitochondrial electron transport cytochrome c to oxygen [GO:0006123]	4.71E-03	4	12	COX6 COX8 COX4 CYC1
	iron assimilation by reduction and transport [GO:0033215]	1.57E-02	2	2	FET3 FTR1
	protein insertion into mitochondrial membrane [GO:0051204]	1.57E-02	2	2	YLH47 MDM38
	ion transport [GO:0006811]	3.35E-02	9	109	CTR3 ATP17 CCC2 ZRT1 AIM14 ATP14 ATP16 FET3 FTR1
	isopentenyl diphosphate biosynthetic process mevalonate pathway [GO:0019287]	3.98E-02	2	3	ERG12 HMG1
	cellular aldehyde metabolic process [GO:0006081]	4.36E-02	3	11	ADH6 AAD4 HFD1

	Category	p-value	NG	NGR	Genes
Group 2	cytoplasmic translation [GO:0002181]	6.80E-34	40	171	RPS29A RPL22A RPL24B RPL26A RPL4B RPL36A RPL37B RPL6B RPS10B RPS11A RPS16A TIF3 RPS21A RPL11B RPL24A RPL4A RPP1A RPS1B RPL19A RPL7A RPL43B RPL33B RPS10A RPL12B RPL31B RPL11A RPL20B RPL18A RPL23B RPL9B RPS28B RPS13 RPS29B RPS26B DPS1 RPL42A RPL35A RPL27B RPS30A RPP2A
	translation [GO:0006412]	1.74E-14	28	242	RPS29A RPL22A RPL24B RPL26A RPL4B RPL36A RPL37B RPL6B TIF3 RPS21A RPL11B FRS1 RPL24A RPL4A RPS1B RPL7A GRS1 RPL33B RPL31B RPL11A RPL9B RPS28B RPS13 RPS29B RPS26B DPS1 RPL27B PRT1
	rRNA export from nucleus [GO:0006407)	4.69E-03	5	27	RPS10B RPS10A RPS28B RPS26B RNA1
	pyridoxal 5'-phosphate salvage [GO:0009443]	1.31E-02	2	2	BUD17 BUD16
Group 3	arginine biosynthetic process [GO:0006526]	1.69E-06	5	10	ARG3 CPA2 ARG1 ARG8 ARG7
	retrograde transport, endosome to Golgi [GO:0042147]	2.37E-02	3	18	VPS54 VPS17 VPS51
	ornithine biosynthetic process [GO:0006592]	3.27E-02	2	4	ARG8 ARG7
	deoxyribonucleotide biosynthetic process [GO:0009263]	4.03E-02	2	6	RNR4 RNR3
	cytogamy [GO:0000755]	4.96E-02	2	8	FUS1 FIG1
	cellular amino acid biosynthetic process [GO:0008652]	4.97E-02	5	98	ARG3 CPA2 ARG1 ARG8 ARG7
Group 4	transcription, DNA-dependent [GO:0006351]	7.00E-03	18	540	RPN4 IWR1 ARG82 VPS72 JHD1 DOT6 TOS8 RTF1 RTT102 BYE1 MSN4 RSF1 GAT2 CAT8 BDP1 AHC1 LGE1 CSR2
	regulation of transcription, DNA-dependent [GO:0006355]	8.28E-03	17	507	RPN4 IWR1 ARG82 JHD1 DOT6 TOS8 RTF1 RTT102 BYE1 MSN4 RSF1 GAT2 CAT8 BDP1 AHC1 LGE1 CSR2
