# Supplementary material for: Key role of lipid management in nitrogen and aroma metabolism in an evolved wine yeast strain
Source: Microb Cell Fact. 2016 Feb 9;15:32. doi: 10.1186/s12934-016-0434-6 (PMC4748530; doi:10.1186/s12934-016-0434-6)
Supplement: Supplementary file 4 — 10.1186/s12934-016-0434-6 Genes differentially expressed between the two sampling times for Affinity™ ECA5. [file 12934_2016_434_MOESM4_ESM.pdf]

Systematic name	Functional name	logFC	FC	adj.P.Val
YPR065W	ROX1	-2.428	0.186	2.11E-07
YBL043W	ECM13	-2.390	0.191	3.05E-04
YAR064W	YAR064W	-2.262	0.209	3.28E-02
YMR317W	YMR317W	-2.138	0.227	1.33E-08
YFL051C	YFL051C	-1.997	0.251	2.19E-06
YDL039C	PRM7	-1.976	0.254	1.34E-07
YLL012W	YEH1	-1.972	0.255	8.17E-05
YMR070W	MOT3	-1.969	0.255	3.22E-03
YHR213W	YHR213W	-1.870	0.274	1.71E-05
YHR213W	YHR213W	-1.866	0.274	2.45E-03
YOR192C	THI72	-1.823	0.283	2.76E-08
YLR037C	PAU23	-1.719	0.304	7.70E-05
YJR048W	CYC1	-1.708	0.306	1.93E-04
YML075C	HMG1	-1.673	0.314	1.05E-03
YLL025W	PAU17	-1.632	0.323	7.36E-04
YIR031C	DAL7	-1.625	0.324	8.23E-04
YNL331C	AAD14	-1.604	0.329	1.15E-05
YNL231C	PDR16	-1.597	0.331	1.41E-03
YDR492W	IZH1	-1.533	0.345	1.03E-04
YPL117C	IDI1	-1.528	0.347	2.75E-05
YGR065C	VHT1	-1.523	0.348	2.13E-05
YMR325W	PAU19	-1.523	0.348	2.11E-07
YOL161C	PAU20	-1.521	0.349	2.36E-04
YDL236W	PHO13	-1.517	0.350	1.73E-04
YDL037C	BSC1	-1.506	0.352	9.69E-06
YBR242W	YBR242W	-1.486	0.357	1.49E-03
YIR041W	PAU15	-1.459	0.364	4.40E-05
YNL024C	YNL024C	-1.428	0.372	8.12E-03
YNR057C	BIO4	-1.403	0.378	5.53E-05
YOR062C	YOR062C	-1.396	0.380	1.10E-05
YMR015C	ERG5	-1.395	0.380	3.55E-04
YKL224C	PAU16	-1.394	0.381	9.18E-03
YHR092C	HXT4	-1.376	0.385	1.23E-04
YOR394W	PAU21	-1.374	0.386	2.07E-04
YCR104W	PAU3	-1.368	0.387	1.29E-03
YER060W-A	FCY22	-1.366	0.388	3.68E-05
YML126C	ERG13	-1.362	0.389	9.13E-06
YLR153C	ACS2	-1.360	0.390	2.71E-05
YNL111C	CYB5	-1.355	0.391	5.07E-06
YGL261C	PAU11	-1.341	0.395	1.08E-05
YPL282C	PAU22	-1.329	0.398	3.40E-04
YMR220W	ERG8	-1.329	0.398	8.93E-05
YAL068C	PAU8	-1.325	0.399	4.94E-07
YHR039C	MSC7	-1.318	0.401	4.58E-06
YDR542W	PAU10	-1.312	0.403	2.59E-05

EC1118_1O4_6612g	EC1118_1O4_6612g	-1.312	0.403	4.70E-05
YER028C	MIG3	-1.284	0.411	2.99E-07
YNL280C	ERG24	-1.270	0.415	1.08E-03
YLR089C	ALT1	-1.263	0.417	6.75E-06
YDL141W	BPL1	-1.260	0.417	2.40E-06
YLR237W	THI7	-1.257	0.418	3.81E-07
YFL034C-A	RPL22B	-1.253	0.420	1.99E-06
YMR134W	ERG29	-1.251	0.420	5.49E-06
YOL029C	YOL029C	-1.250	0.421	1.33E-07
YLR149C-A	YLR149C-A	-1.240	0.423	1.34E-04
YGR286C	BIO2	-1.232	0.426	1.22E-05
YDR222W	YDR222W	-1.202	0.435	6.53E-04
YOL028C	YAP7	-1.194	0.437	5.87E-05
YER060W	FCY21	-1.191	0.438	8.98E-06
YML008C	ERG6	-1.191	0.438	2.05E-05
YOR051C	ETT1	-1.164	0.446	3.29E-04
YLR372W	SUR4	-1.161	0.447	6.02E-03
YIL176C	PAU14	-1.156	0.449	7.18E-03
YHL046C	PAU13	-1.156	0.449	1.96E-02
YOL159C	YOL159C	-1.153	0.450	2.07E-06
YKR075C	YKR075C	-1.143	0.453	5.13E-05
YNL141W	AAH1	-1.104	0.465	1.26E-02
YLR175W	CBF5	-1.103	0.466	1.10E-03
YOR361C	PRT1	-1.095	0.468	5.83E-03
YNR076W	PAU6	-1.090	0.470	1.09E-03
YOR182C	RPS30B	-1.089	0.470	1.05E-02
YJL025W	RRN7	-1.086	0.471	5.55E-07
YIR030C	DCG1	-1.084	0.472	5.75E-05
YDR541C	YDR541C	-1.077	0.474	1.70E-05
YHR180W	YHR180W	-1.067	0.477	1.40E-04
YHL026C	YHL026C	-1.065	0.478	2.50E-07
YLR406C	RPL31B	-1.065	0.478	7.17E-06
YBL108C-A	PAU9	-1.062	0.479	1.30E-02
YCR020C	PET18	-1.062	0.479	8.65E-03
YGL223C	COG1	-1.057	0.481	4.58E-05
YGR085C	RPL11B	-1.054	0.482	1.50E-04
YDL208W	NHP2	-1.053	0.482	5.04E-04
YHR094C	HXT1	-1.047	0.484	6.96E-03
YDR165W	TRM82	-1.043	0.485	8.14E-05
YNR065C	YNR065C	-1.041	0.486	8.89E-04
YGR036C	CAX4	-1.041	0.486	3.25E-04
YPL245W	YPL245W	-1.036	0.488	3.86E-02
YNR018W	RCF2	-1.034	0.488	2.52E-03
YIL145C	PAN6	-1.032	0.489	7.10E-04
YJL191W	RPS14B	-1.028	0.490	3.11E-05
YGR175C	ERG1	-1.024	0.492	9.85E-03

YDR284C	DPP1	-1.024	0.492	4.72E-03
YGR177C	ATF2	-1.021	0.493	4.20E-05
YHL043W	ECM34	-1.020	0.493	3.62E-04
YDR294C	DPL1	-1.020	0.493	3.11E-02
YLR461W	PAU4	-1.016	0.495	5.11E-03
YIL123W	SIM1	-1.009	0.497	1.74E-02
YGR078C	PAC10	-1.009	0.497	2.38E-04
YKL156W	RPS27A	-1.006	0.498	2.27E-03
YCR105W	ADH7	-1.005	0.498	2.75E-02
YHR019C	DED81	-1.004	0.499	2.34E-02
YGL101W	YGL101W	-0.999	0.500	1.23E-03
YBL081W	YBL081W	-0.999	0.500	1.68E-03
YBR084W	MIS1	-0.993	0.502	3.31E-05
YNR056C	BIO5	-0.986	0.505	1.91E-02
YDL244W	THI13	-0.983	0.506	1.24E-05
YPL198W	RPL7B	-0.978	0.508	1.44E-03
YPL189W	GUP2	-0.977	0.508	4.91E-04
YPL252C	YAH1	-0.977	0.508	1.70E-05
YLR287C-A	RPS30A	-0.975	0.509	1.79E-02
YNL046W	YNL046W	-0.973	0.510	2.38E-04
YAR068W	YAR068W	-0.963	0.513	2.59E-02
YGL253W	HXK2	-0.959	0.514	2.85E-03
YMR049C	ERB1	-0.958	0.515	6.47E-04
YLR367W	RPS22B	-0.951	0.517	3.35E-02
YBL068W	PRS4	-0.948	0.518	9.57E-04
YJL198W	PHO90	-0.948	0.519	3.87E-03
YBL053W	YBL053W	-0.945	0.519	3.03E-02
YNR046W	TRM112	-0.945	0.519	3.67E-02
YLR197W	NOP56	-0.945	0.520	1.03E-02
YGR049W	SCM4	-0.940	0.521	2.60E-04
YFR056C	YFR056C	-0.934	0.523	9.12E-04
YAR068W	YAR068W	-0.934	0.523	2.03E-03
YIL128W	MET18	-0.933	0.524	4.33E-03
YNR019W	ARE2	-0.931	0.525	1.27E-02
YEL065W	SIT1	-0.929	0.525	3.55E-03
YEL034W	HYP2	-0.928	0.525	2.26E-02
YOR107W	RGS2	-0.928	0.526	2.32E-03
YDL081C	RPP1A	-0.928	0.526	2.33E-02
YOR203W	YOR203W	-0.927	0.526	6.02E-05
YJR070C	LIA1	-0.926	0.526	1.64E-02
YER019W	ISC1	-0.924	0.527	1.29E-03
YDR324C	UTP4	-0.923	0.528	3.56E-04
YNL182C	IPI3	-0.923	0.528	5.32E-05
YPL093W	NOG1	-0.921	0.528	4.24E-05
YIL016W	SNL1	-0.919	0.529	7.23E-06
YHR089C	GAR1	-0.914	0.531	7.43E-05

YAL029C	MYO4	-0.910	0.532	1.55E-02
YJL208C	NUC1	-0.910	0.532	7.71E-04
YMR194W	RPL36A	-0.909	0.532	4.79E-05
YOL093W	TRM10	-0.908	0.533	6.47E-03
YIL091C	UTP25	-0.906	0.534	2.84E-03
YLR196W	PWP1	-0.906	0.534	4.93E-04
YNL128W	TEP1	-0.904	0.534	2.82E-02
YNL061W	NOP2	-0.903	0.535	2.37E-05
YOR222W	ODC2	-0.902	0.535	2.64E-04
YHL042W	YHL042W	-0.901	0.536	1.69E-02
YLR130C	ZRT2	-0.901	0.536	4.97E-03
YDL169C	UGX2	0.900	1.867	6.05E-04
YGR122C-A	YGR122C-A	0.903	1.870	4.62E-04
YDL181W	INH1	0.903	1.870	5.71E-03
YMR291W	TDA1	0.906	1.874	3.22E-03
YLR126C	YLR126C	0.906	1.874	5.50E-03
YMR031C	EIS1	0.907	1.875	4.93E-04
YOL023W	IFM1	0.908	1.877	8.12E-03
YGR254W	ENO1	0.909	1.877	1.67E-04
YJL221C	IMA4	0.909	1.878	7.52E-04
YML133C	YML133C	0.913	1.883	1.55E-02
YOR288C	MPD1	0.916	1.887	9.30E-05
YLL020C	YLL020C	0.917	1.889	1.13E-06
YMR020W	FMS1	0.922	1.895	5.43E-03
YMR188C	MRPS17	0.928	1.902	3.29E-04
YPL278C	YPL278C	0.928	1.903	1.67E-02
YML128C	MSC1	0.928	1.903	4.91E-04
YMR053C	STB2	0.928	1.903	6.14E-04
YLR107W	REX3	0.930	1.905	1.90E-02
YDL019C	OSH2	0.931	1.906	1.80E-05
YOR075W	UFE1	0.931	1.907	4.10E-04
YLR467W	YRF1-5	0.934	1.911	9.12E-03
YGL045W	RIM8	0.943	1.923	1.70E-03
YHR139C	SPS100	0.944	1.924	1.04E-05
YLR466W	YRF1-4	0.944	1.925	2.39E-05
YJR103W	URA8	0.948	1.929	2.45E-03
YNL092W	YNL092W	0.950	1.932	6.44E-03
YLL066C	YLL066C	0.951	1.933	8.08E-04
YIR017C	MET28	0.951	1.934	1.42E-02
YIL172C	IMA3	0.952	1.934	1.38E-02
YJR008W	MHO1	0.952	1.935	2.32E-04
YOR347C	PYK2	0.957	1.941	6.41E-04
YMR085W	YMR085W	0.959	1.944	2.47E-04
YDR255C	RMD5	0.965	1.952	7.25E-03
YMR315W	YMR315W	0.965	1.952	2.36E-03
YDR485C	VPS72	0.967	1.954	3.07E-03

YOL157C	IMA2	0.969	1.957	8.00E-04
YLR109W	AHP1	0.971	1.960	2.03E-03
YPL055C	LGE1	0.974	1.964	7.43E-03
YOR195W	SLK19	0.974	1.965	1.31E-03
YKL124W	SSH4	0.980	1.972	1.27E-03
YDR532C	KRE28	0.983	1.976	8.35E-05
YHR153C	SPO16	0.983	1.977	4.02E-02
YLR350W	ORM2	0.984	1.978	1.90E-02
YJL155C	FBP26	0.988	1.984	1.94E-05
YOR389W	YOR389W	0.989	1.984	4.82E-04
YIL136W	OM45	0.989	1.985	5.36E-04
YLR054C	OSW2	0.989	1.985	3.07E-03
YJL219W	HXT9	0.989	1.985	4.95E-03
YGR032W	GSC2	0.990	1.986	1.59E-04
YPR026W	ATH1	0.993	1.990	4.78E-03
YJL149W	DAS1	0.996	1.994	5.23E-05
YER002W	NOP16	0.998	1.997	3.07E-02
YMR030W	RSF1	1.002	2.003	2.86E-03
YIR038C	GTT1	1.005	2.006	4.56E-03
YMR271C	URA10	1.005	2.006	8.01E-03
YCR005C	CIT2	1.011	2.016	8.80E-06
YDR247W	VHS1	1.020	2.028	1.21E-04
YGR070W	ROM1	1.020	2.028	1.88E-04
YMR170C	ALD2	1.020	2.028	3.04E-02
YAL028W	FRT2	1.024	2.034	2.07E-05
YNL014W	HEF3	1.030	2.042	3.07E-02
YNL007C	SIS1	1.031	2.044	3.05E-03
YKL071W	YKL071W	1.031	2.044	1.80E-05
YER079W	YER079W	1.032	2.045	5.16E-05
YGR102C	GTF1	1.034	2.048	2.56E-03
YJL141C	YAK1	1.042	2.059	5.53E-05
YER162C	RAD4	1.043	2.060	8.57E-07
YER037W	PHM8	1.044	2.062	1.57E-02
YIL050W	PCL7	1.045	2.063	5.04E-04
YER088C	DOT6	1.045	2.064	4.14E-04
YML118W	NGL3	1.047	2.067	4.93E-04
YBR126C	TPS1	1.048	2.067	3.55E-02
YEL039C	CYC7	1.051	2.072	7.41E-05
YLR120C	YPS1	1.052	2.074	2.57E-02
YGL096W	TOS8	1.062	2.088	2.78E-04
YKL091C	YKL091C	1.064	2.090	8.24E-07
YOR134W	BAG7	1.065	2.093	1.74E-05
YDL206W	YDL206W	1.067	2.095	2.16E-03
YLR151C	PCD1	1.067	2.095	8.38E-03
EC1118_1F14_0089g	EC1118_1F14_0089g	1.068	2.096	1.37E-04
YMR114C	YMR114C	1.069	2.098	3.88E-06

YHR087W	RTC3	1.069	2.099	5.18E-03
YKL070W	YKL070W	1.072	2.102	3.71E-03
YGL191W	COX13	1.072	2.103	2.75E-05
YML132W	COS3	1.074	2.106	2.21E-03
YLR149C	YLR149C	1.078	2.111	2.52E-06
YOR306C	MCH5	1.079	2.112	1.16E-03
YLR247C	IRC20	1.081	2.115	1.08E-05
YDR069C	DOA4	1.084	2.120	2.14E-06
YAL062W	GDH3	1.085	2.122	9.71E-03
YJL049W	YJL049W	1.086	2.124	3.44E-03
YDR313C	PIB1	1.088	2.126	1.19E-02
YGL005C	COG7	1.089	2.127	8.67E-05
YIL144W	NDC80	1.093	2.133	9.84E-06
YBR302C	COS2	1.096	2.138	3.99E-07
YHR160C	PEX18	1.097	2.138	1.49E-02
YMR311C	GLC8	1.098	2.140	3.40E-05
YDR516C	EMI2	1.098	2.141	3.58E-05
YKL124W	SSH4	1.099	2.142	1.75E-05
YMR306W	FKS3	1.104	2.149	4.78E-02
YHR097C	YHR097C	1.104	2.149	5.67E-03
YBR147W	RTC2	1.110	2.158	2.58E-02
YDL123W	SNA4	1.118	2.171	5.00E-04
YOR019W	YOR019W	1.118	2.171	4.75E-06
YGR053C	YGR053C	1.121	2.176	3.11E-05
YNL305C	BXI1	1.122	2.177	3.87E-04
YJL163C	YJL163C	1.123	2.178	2.70E-03
YBL060W	YEL1	1.129	2.187	5.81E-04
YBR214W	SDS24	1.133	2.193	3.44E-03
YBL086C	YBL086C	1.141	2.206	2.84E-04
YOR185C	GSP2	1.145	2.211	3.56E-03
YBR285W	YBR285W	1.146	2.213	4.48E-03
YDR262W	YDR262W	1.146	2.213	1.04E-06
YPL230W	USV1	1.161	2.236	3.20E-04
YBR287W	YBR287W	1.163	2.239	4.79E-05
YJL142C	IRC9	1.172	2.253	2.95E-02
YCR091W	KIN82	1.173	2.254	3.82E-05
YNL173C	MDG1	1.173	2.254	4.58E-07
YBR298C	MAL31	1.174	2.256	6.02E-05
YMR174C	PAI3	1.175	2.258	3.33E-02
YPR184W	GDB1	1.175	2.259	5.12E-06
YMR250W	GAD1	1.179	2.264	3.07E-03
YLL055W	YCT1	1.180	2.266	9.26E-06
YER053C	PIC2	1.181	2.268	1.53E-02
YDL020C	RPN4	1.183	2.270	2.47E-06
YOR036W	PEP12	1.186	2.275	7.58E-06
YKR049C	FMP46	1.186	2.276	6.02E-05

YPL017C	IRC15	1.188	2.278	4.78E-03
YLR010C	TEN1	1.189	2.279	1.40E-04
YKR058W	GLG1	1.189	2.280	1.94E-05
YJL094C	KHA1	1.190	2.281	4.46E-05
YDR216W	ADR1	1.190	2.281	1.91E-02
YBR290W	BSD2	1.194	2.288	2.07E-05
YIL097W	FYV10	1.195	2.290	2.47E-03
YDR070C	FMP16	1.205	2.305	1.13E-04
YDR453C	TSA2	1.205	2.305	7.48E-06
YHL036W	MUP3	1.210	2.314	8.92E-05
YMR100W	MUB1	1.211	2.315	1.69E-02
YPL164C	MLH3	1.212	2.317	9.66E-06
YAL060W	BDH1	1.222	2.333	3.36E-07
YAL017W	PSK1	1.223	2.335	1.91E-02
YGR023W	MTL1	1.229	2.345	1.48E-03
YMR090W	YMR090W	1.231	2.347	5.32E-05
YLR138W	NHA1	1.233	2.350	5.94E-03
YNL136W	EAF7	1.235	2.353	1.02E-03
YDL010W	GRX6	1.235	2.354	1.09E-04
EC1118_1O4_6579g	EC1118_1O4_6579g	1.243	2.367	3.38E-04
YGL180W	ATG1	1.245	2.369	2.34E-03
YBL064C	PRX1	1.247	2.373	4.62E-04
YDL234C	GYP7	1.251	2.379	2.91E-03
YPR085C	ASA1	1.252	2.382	5.89E-07
YIL098C	FMC1	1.254	2.385	2.15E-06
YDR185C	UPS3	1.258	2.392	1.52E-02
YKL142W	MRP8	1.260	2.394	9.05E-05
YIL024C	YIL024C	1.267	2.407	4.25E-04
YIR014W	YIR014W	1.272	2.416	7.94E-04
YOR223W	YOR223W	1.274	2.418	2.14E-06
YBR068C	BAP2	1.274	2.419	1.53E-06
YBR169C	SSE2	1.284	2.435	1.74E-03
YDR273W	DON1	1.286	2.438	4.58E-07
YER103W	SSA4	1.290	2.445	1.14E-03
YOL109W	ZEO1	1.306	2.473	1.03E-02
YHL021C	AIM17	1.308	2.477	9.87E-07
YMR196W	YMR196W	1.311	2.481	2.59E-03
YGR030C	POP6	1.328	2.510	4.68E-05
YFR040W	SAP155	1.335	2.523	2.14E-04
YDL079C	MRK1	1.336	2.524	3.37E-04
YGR225W	AMA1	1.345	2.540	1.66E-04
YER142C	MAG1	1.350	2.550	1.17E-05
YDR473C	PRP3	1.357	2.561	5.63E-04
YIL101C	XBP1	1.357	2.561	1.88E-05
YOL154W	ZPS1	1.363	2.573	4.79E-05
YJL102W	MEF2	1.386	2.613	5.73E-07

YPL240C	HSP82	1.394	2.628	1.50E-06
YML034W	SRC1	1.395	2.630	1.13E-04
YDL173W	PAR32	1.397	2.634	8.61E-04
YLR177W	YLR177W	1.398	2.635	1.66E-04
YDL115C	IWR1	1.399	2.637	1.98E-04
YBR066C	NRG2	1.402	2.642	8.22E-04
YJL164C	TPK1	1.402	2.642	4.97E-06
YNL142W	MEP2	1.405	2.648	9.40E-05
YOR052C	TMC1	1.412	2.662	2.13E-04
YKL026C	GPX1	1.416	2.669	8.07E-04
YJR061W	YJR061W	1.422	2.680	2.11E-07
YPL119C	DBP1	1.424	2.683	8.68E-07
YLR178C	TFS1	1.448	2.729	3.75E-02
YPR154W	PIN3	1.455	2.742	2.45E-03
YOR049C	RSB1	1.460	2.750	6.72E-04
YNL212W	VID27	1.460	2.751	1.04E-03
YKL151C	YKL151C	1.473	2.775	1.83E-05
YLR152C	YLR152C	1.476	2.782	1.00E-07
YCL040W	GLK1	1.480	2.790	2.14E-06
YMR136W	GAT2	1.485	2.799	3.74E-06
YAL055W	PEX22	1.485	2.800	6.82E-05
YMR206W	YMR206W	1.492	2.813	2.27E-03
YGR201C	YGR201C	1.493	2.814	1.64E-05
YNL015W	PBI2	1.496	2.820	1.83E-05
YPL201C	YIG1	1.500	2.829	2.47E-03
YOR120W	GCY1	1.505	2.838	2.39E-07
YDR179C	CSN9	1.508	2.844	2.19E-06
YBR284W	YBR284W	1.516	2.859	4.70E-05
YKR034W	DAL80	1.519	2.866	1.10E-03
YLR338W	OPI9	1.535	2.897	1.22E-04
YNL194C	YNL194C	1.539	2.907	3.28E-08
YMR135C	GID8	1.542	2.913	2.59E-03
YHR140W	YHR140W	1.546	2.921	2.80E-08
YOL016C	CMK2	1.549	2.927	2.88E-03
YIL119C	RPI1	1.554	2.936	5.61E-06
YCR039C	MATALPHA2	1.556	2.940	3.32E-07
YCL067C	HMLALPHA2	1.560	2.949	2.43E-05
YPR101W	SNT309	1.562	2.952	1.55E-04
YOR173W	DCS2	1.570	2.969	2.32E-04
YDL024C	DIA3	1.573	2.976	7.08E-07
YGL184C	STR3	1.580	2.990	3.42E-05
YHR146W	CRP1	1.602	3.036	8.96E-07
YKR014C	YPT52	1.609	3.051	8.39E-05
YJL153C	INO1	1.615	3.062	1.34E-07
YJR152W	DAL5	1.620	3.074	2.82E-07
YMR040W	YET2	1.653	3.144	1.89E-07

YKL035W	UGP1	1.653	3.144	7.00E-06
YNL077W	APJ1	1.690	3.227	1.43E-05
YDL124W	YDL124W	1.702	3.254	7.58E-08
YJL034W	KAR2	1.714	3.280	2.45E-03
YDR258C	HSP78	1.721	3.297	4.27E-08
YOR257W	CDC31	1.723	3.302	3.14E-06
YHR138C	YHR138C	1.726	3.309	1.27E-03
YNL093W	YPT53	1.729	3.316	2.47E-07
YJL166W	QCR8	1.764	3.396	8.68E-07
YDL210W	UGA4	1.777	3.428	4.93E-04
YLR312C	YLR312C	1.829	3.552	7.03E-06
YAL061W	BDH2	1.840	3.579	9.17E-06
YEL060C	PRB1	1.858	3.625	1.93E-04
YMR105C	PGM2	1.881	3.682	2.31E-05
YDR533C	HSP31	1.911	3.760	1.01E-05
EC1118_1O4_6667g	EC1118_1O4_6667g	1.919	3.781	2.20E-07
EC1118_1O4_6656g	EC1118_1O4_6656g	1.988	3.967	1.62E-05
YGR008C	STF2	1.993	3.980	8.35E-06
YDR173C	ARG82	2.004	4.011	3.59E-07
YER054C	GIP2	2.029	4.082	3.42E-05
YJL144W	YJL144W	2.047	4.131	7.58E-08
YNL196C	SLZ1	2.064	4.182	9.91E-08
YMR107W	SPG4	2.093	4.267	3.82E-02
YIL045W	PIG2	2.106	4.306	1.03E-03
YGR142W	BTN2	2.113	4.325	3.03E-02
YPL186C	UIP4	2.113	4.327	4.94E-07
YAL005C	SSA1	2.116	4.334	5.95E-07
YOL052C-A	DDR2	2.130	4.376	8.85E-06
YPR030W	CSR2	2.157	4.461	3.23E-04
YPR160W	GPH1	2.174	4.513	5.56E-09
YMR251W-A	HOR7	2.183	4.542	7.08E-07
YML100W	TSL1	2.188	4.557	3.54E-06
YEL012W	UBC8	2.192	4.569	2.99E-08
YGR161C	RTS3	2.221	4.661	3.04E-02
YFR015C	GSY1	2.347	5.087	1.00E-07
YDR171W	HSP42	2.348	5.092	1.40E-07
YDR043C	NRG1	2.407	5.305	2.99E-08
YLR258W	GSY2	2.475	5.561	7.66E-07
YNR034W-A	YNR034W-A	2.526	5.761	3.56E-04
YAL054C	ACS1	2.536	5.799	1.65E-09
YLR327C	TMA10	2.602	6.073	3.46E-08
YER067W	RGI1	2.687	6.438	2.46E-06
YFR017C	IGD1	2.693	6.467	4.23E-09
YEL011W	GLC3	2.735	6.660	9.23E-09
YBR072W	HSP26	2.870	7.312	8.06E-05
YER150W	SPI1	2.907	7.501	1.65E-09

YMR081C	ISF1	2.984	7.914	3.28E-08
YCR098C	GIT1	3.152	8.891	4.23E-09
YCR021C	HSP30	3.192	9.137	3.22E-07
YOR178C	GAC1	3.547	11.685	5.26E-03
YDR342C	HXT7	3.566	11.843	4.23E-09
YOR348C	PUT4	3.807	13.999	5.56E-09
YDR343C	HXT6	3.971	15.684	1.09E-05
