# Supplementary material for: Key role of lipid management in nitrogen and aroma metabolism in an evolved wine yeast strain
Source: Microb Cell Fact. 2016 Feb 9;15:32. doi: 10.1186/s12934-016-0434-6 (PMC4748530; doi:10.1186/s12934-016-0434-6)
Supplement: Supplementary file 5 — 10.1186/s12934-016-0434-6 Genes differentially expressed between Affinity™ ECA5 and Lalvin EC1118® for the first time of sampling. [file 12934_2016_434_MOESM5_ESM.pdf]

Systematic name	Functional name	logFC	FC	adj.P.Val
YMR175W	SIP18	-4.297	0.051	9.42E-09
YGR088W	CTT1	-3.742	0.075	1.65E-09
YFL014W	HSP12	-3.630	0.081	1.50E-10
YML054C	CYB2	-3.549	0.085	6.80E-12
YMR169C	ALD3	-3.522	0.087	2.37E-08
YGR201C	YGR201C	-3.205	0.108	4.28E-09
YPL223C	GRE1	-3.198	0.109	7.44E-07
YGL255W	ZRT1	-3.178	0.111	7.33E-07
YMR174C	PAI3	-3.170	0.111	1.96E-05
YBR116C	YBR116C	-2.976	0.127	1.19E-06
YPL036W	PMA2	-2.962	0.128	4.72E-07
YDR270W	CCC2	-2.945	0.130	1.74E-08
YNL112W	DBP2	-2.941	0.130	3.08E-08
YMR244W	YMR244W	-2.907	0.133	6.96E-03
YER145C	FTR1	-2.828	0.141	9.86E-09
YGR052W	FMP48	-2.758	0.148	1.30E-08
YDL223C	HBT1	-2.711	0.153	7.52E-07
YER053C-A	YER053C-A	-2.693	0.155	6.21E-08
YPR160W	GPH1	-2.651	0.159	8.08E-10
YCL036W	GFD2	-2.634	0.161	4.11E-09
YML075C	HMG1	-2.510	0.176	4.14E-05
YJL025W	RRN7	-2.471	0.180	4.14E-11
YPR151C	SUE1	-2.468	0.181	4.51E-10
YGR256W	GND2	-2.430	0.186	3.61E-04
YDR453C	TSA2	-2.401	0.189	4.28E-09
YML128C	MSC1	-2.379	0.192	2.93E-08
YGR043C	NQM1	-2.376	0.193	2.18E-05
YDL181W	INH1	-2.312	0.201	1.04E-06
YGR066C	YGR066C	-2.302	0.203	6.21E-08
YBR117C	TKL2	-2.292	0.204	3.91E-02
YNL237W	YTP1	-2.274	0.207	3.84E-08
YJR048W	CYC1	-2.272	0.207	2.09E-05
YMR250W	GAD1	-2.267	0.208	1.20E-05
YPR193C	HPA2	-2.236	0.212	7.48E-07
YNL052W	COX5A	-2.226	0.214	1.08E-08
YMR090W	YMR090W	-2.218	0.215	1.40E-07
YLR395C	COX8	-2.216	0.215	1.13E-07
YOR062C	YOR062C	-2.216	0.215	1.13E-07
YGR248W	SOL4	-2.209	0.216	8.54E-07
YLR168C	UPS2	-2.174	0.222	8.08E-10
YNL270C	ALP1	-2.144	0.226	9.42E-09
YGR065C	VHT1	-2.133	0.228	9.91E-07
YPR184W	GDB1	-2.127	0.229	9.42E-09
YPR061C	JID1	-2.125	0.229	5.97E-07
YDR492W	IZH1	-2.119	0.230	7.45E-06

YDR070C	FMP16	-2.116	0.231	4.25E-07
YNR053C	NOG2	-2.099	0.233	1.93E-05
YHR001W-A	QCR10	-2.092	0.234	1.94E-05
YFR017C	IGD1	-2.088	0.235	1.32E-07
YPL280W	HSP32	-2.071	0.238	9.42E-09
YMR105C	PGM2	-2.068	0.239	1.82E-05
YGL187C	COX4	-2.063	0.239	3.68E-08
YOL052C-A	DDR2	-2.060	0.240	2.70E-05
YKL187C	FAT3	-2.043	0.243	4.81E-05
YMR322C	SNO4	-2.040	0.243	6.25E-08
YPR065W	ROX1	-2.019	0.247	4.18E-06
YNR018W	RCF2	-2.014	0.248	8.05E-06
YPL274W	SAM3	-2.007	0.249	1.32E-08
YEL039C	CYC7	-1.994	0.251	1.13E-07
YGR079W	YGR079W	-1.976	0.254	1.98E-07
YMR256C	COX7	-1.976	0.254	4.38E-07
YBR230C	OM14	-1.973	0.255	9.61E-06
YBR072W	HSP26	-1.966	0.256	5.00E-03
YDR533C	HSP31	-1.966	0.256	1.63E-05
YIR038C	GTT1	-1.964	0.256	1.77E-05
YNL111C	CYB5	-1.963	0.257	1.35E-07
YOR338W	YOR338W	-1.945	0.260	4.29E-04
YDR222W	YDR222W	-1.943	0.260	1.04E-05
YOR173W	DCS2	-1.941	0.260	5.63E-05
YGL191W	COX13	-1.939	0.261	6.21E-08
YBR183W	YPC1	-1.922	0.264	3.06E-05
YPL281C	ERR2	-1.918	0.265	5.81E-06
YKL026C	GPX1	-1.903	0.267	9.80E-05
YGR182C	YGR182C	-1.900	0.268	2.90E-07
YML043C	RRN11	-1.887	0.270	2.39E-07
YMR070W	MOT3	-1.877	0.272	8.66E-03
YJL116C	NCA3	-1.843	0.279	8.54E-07
YEL024W	RIP1	-1.843	0.279	3.77E-07
YJL048C	UBX6	-1.827	0.282	8.47E-05
YFL030W	AGX1	-1.814	0.284	5.07E-04
YBL030C	PET9	-1.805	0.286	7.37E-07
YOR374W	ALD4	-1.804	0.286	1.62E-04
YKR046C	PET10	-1.786	0.290	2.59E-07
YNL141W	AAH1	-1.784	0.290	5.81E-04
YKR049C	FMP46	-1.781	0.291	1.41E-06
YMR015C	ERG5	-1.777	0.292	6.48E-05
YOR393W	ERR1	-1.766	0.294	8.54E-07
YKL078W	DHR2	-1.752	0.297	6.17E-06
YBR242W	YBR242W	-1.750	0.297	7.45E-04
YCR061W	YCR061W	-1.750	0.297	5.65E-03
YNL269W	BSC4	-1.738	0.300	6.72E-06

YOR317W	FAA1	-1.735	0.300	3.08E-08
YAL061W	BDH2	-1.729	0.302	3.91E-05
YNL200C	YNL200C	-1.727	0.302	1.98E-07
YBL042C	FUI1	-1.726	0.302	8.54E-07
YAL025C	MAK16	-1.720	0.304	2.15E-07
YDR223W	CRF1	-1.719	0.304	3.28E-05
YBL029W	YBL029W	-1.701	0.308	3.36E-07
YDL204W	RTN2	-1.695	0.309	1.04E-05
YCR072C	RSA4	-1.694	0.309	3.90E-07
YER053C	PIC2	-1.686	0.311	2.20E-03
YKL107W	YKL107W	-1.685	0.311	1.43E-05
YOL151W	GRE2	-1.671	0.314	1.87E-07
YOR391C	HSP33	-1.662	0.316	7.75E-05
YPL061W	ALD6	-1.656	0.317	1.27E-04
YPR191W	QCR2	-1.652	0.318	5.20E-04
YLL009C	COX17	-1.646	0.320	2.33E-05
YDL124W	YDL124W	-1.641	0.321	2.15E-07
YKL150W	MCR1	-1.638	0.321	1.13E-05
YEL034W	HYP2	-1.629	0.323	6.76E-04
YGR191W	HIP1	-1.610	0.327	6.31E-07
YER141W	COX15	-1.609	0.328	5.90E-04
YLR109W	AHP1	-1.602	0.329	3.41E-05
YJL166W	QCR8	-1.584	0.334	8.02E-06
YGR225W	AMA1	-1.579	0.335	6.80E-05
YPL186C	UIP4	-1.577	0.335	3.34E-05
YEL011W	GLC3	-1.575	0.336	1.23E-05
YKR076W	ECM4	-1.561	0.339	5.73E-04
YNL231C	PDR16	-1.558	0.340	3.36E-03
EC1118_1O4_6546g	EC1118_1O4_6546g	-1.554	0.340	4.08E-05
YDL085W	NDE2	-1.549	0.342	2.24E-03
YDR299W	BFR2	-1.542	0.343	6.56E-06
YMR002W	MIX17	-1.538	0.344	5.41E-06
YPR098C	YPR098C	-1.533	0.346	1.29E-06
YML042W	CAT2	-1.533	0.346	9.87E-04
YMR068W	AVO2	-1.524	0.348	1.10E-05
YCR057C	PWP2	-1.514	0.350	2.38E-04
YGR049W	SCM4	-1.514	0.350	3.59E-06
YHR066W	SSF1	-1.510	0.351	1.86E-03
YDR504C	SPG3	-1.505	0.352	1.18E-04
YOL155C	HPF1	-1.499	0.354	3.35E-03
YBL045C	COR1	-1.499	0.354	1.94E-04
YKL163W	PIR3	-1.496	0.354	3.02E-06
YHR139C	SPS100	-1.493	0.355	1.13E-07
YBR034C	HMT1	-1.486	0.357	3.26E-07
YDL110C	TMA17	-1.485	0.357	4.29E-05
YDR513W	GRX2	-1.483	0.358	5.34E-06

YDR406W	PDR15	-1.481	0.358	8.17E-03
YDR529C	QCR7	-1.478	0.359	1.85E-04
YBL075C	SSA3	-1.475	0.360	2.15E-07
YOL077W-A	ATP19	-1.474	0.360	1.71E-03
YLR258W	GSY2	-1.463	0.363	5.48E-04
YLL012W	YEH1	-1.460	0.364	2.75E-03
YLL008W	DRS1	-1.453	0.365	9.87E-04
YOR287C	RRP36	-1.441	0.368	2.79E-07
YMR316W	DIA1	-1.432	0.371	1.24E-05
YCR005C	CIT2	-1.428	0.372	3.16E-07
YMR302C	YME2	-1.425	0.372	2.11E-06
EC1118_1O4_6557g	EC1118_1O4_6557g	-1.423	0.373	7.24E-03
YNL119W	NCS2	-1.421	0.373	5.32E-07
YGR175C	ERG1	-1.409	0.377	1.69E-03
YDR505C	PSP1	-1.409	0.377	1.62E-06
YIL130W	ASG1	-1.408	0.377	1.04E-05
YGR159C	NSR1	-1.407	0.377	1.10E-03
YMR290C	HAS1	-1.406	0.377	8.68E-06
YPL028W	ERG10	-1.401	0.379	1.31E-03
YBR213W	MET8	-1.400	0.379	6.44E-06
YNL015W	PBI2	-1.396	0.380	8.38E-05
YER103W	SSA4	-1.393	0.381	1.20E-03
YLR038C	COX12	-1.393	0.381	3.10E-03
YLR276C	DBP9	-1.389	0.382	5.65E-06
YOR078W	BUD21	-1.387	0.382	1.05E-06
YLR223C	IFH1	-1.385	0.383	5.72E-06
YHR096C	HXT5	-1.372	0.386	1.20E-03
YLR411W	CTR3	-1.369	0.387	8.60E-06
YGR280C	PXR1	-1.367	0.388	1.14E-03
YLR063W	BMT6	-1.367	0.388	3.17E-05
YOR129C	AFI1	-1.365	0.388	3.47E-04
YJL161W	FMP33	-1.356	0.391	1.32E-05
YGL160W	AIM14	-1.350	0.392	1.40E-06
YDR083W	RRP8	-1.346	0.393	3.14E-03
EC1118_1O4_6612g	EC1118_1O4_6612g	-1.345	0.394	8.00E-05
YLR407W	YLR407W	-1.345	0.394	1.19E-03
YML093W	UTP14	-1.344	0.394	8.59E-07
YNL160W	YGP1	-1.341	0.395	2.31E-03
YHR051W	COX6	-1.340	0.395	5.81E-04
YDR511W	ACN9	-1.339	0.395	3.25E-03
YBR241C	YBR241C	-1.337	0.396	1.84E-04
YHR178W	STB5	-1.333	0.397	9.53E-06
YPL171C	OYE3	-1.333	0.397	2.79E-03
YNL195C	YNL195C	-1.329	0.398	6.42E-04
YHL024W	RIM4	-1.329	0.398	9.18E-04
YMR316C-B	YMR316C-B	-1.325	0.399	1.38E-03

YBR067C	TIP1	-1.319	0.401	9.63E-05
YBR142W	MAK5	-1.318	0.401	3.78E-06
YNL100W	MIC27	-1.304	0.405	1.47E-05
YOR052C	TMC1	-1.304	0.405	9.56E-04
YKL053C-A	MDM35	-1.303	0.405	6.62E-04
YDR496C	PUF6	-1.299	0.406	8.54E-07
YAL005C	SSA1	-1.298	0.407	3.12E-04
YGR110W	CLD1	-1.296	0.407	1.35E-04
YIL087C	AIM19	-1.295	0.407	1.15E-02
YKR087C	OMA1	-1.294	0.408	9.46E-04
YJR104C	SOD1	-1.288	0.410	1.32E-05
YOR051C	ETT1	-1.287	0.410	2.65E-04
YOL084W	PHM7	-1.287	0.410	1.41E-06
YIL169C	YIL169C	-1.280	0.412	6.96E-03
YDL086W	YDL086W	-1.277	0.413	1.76E-03
YER169W	RPH1	-1.276	0.413	1.32E-05
YHR202W	YHR202W	-1.275	0.413	8.60E-06
YHR154W	RTT107	-1.275	0.413	6.19E-05
YDL174C	DLD1	-1.274	0.413	3.30E-05
YOR205C	GEP3	-1.272	0.414	1.16E-03
YBL064C	PRX1	-1.271	0.414	8.25E-04
YMR134W	ERG29	-1.271	0.414	1.03E-05
YDL167C	NRP1	-1.270	0.415	2.40E-03
YHR065C	RRP3	-1.267	0.416	5.94E-05
YBR247C	ENP1	-1.262	0.417	1.13E-05
YNL065W	AQR1	-1.261	0.417	1.12E-04
YDR490C	PKH1	-1.259	0.418	6.28E-05
YHR039C	MSC7	-1.255	0.419	1.71E-05
YIL160C	POT1	-1.253	0.419	7.36E-04
YML126C	ERG13	-1.253	0.420	4.83E-05
YMR272C	SCS7	-1.252	0.420	3.12E-03
YOR359W	VTS1	-1.252	0.420	2.11E-05
YJL112W	MDV1	-1.248	0.421	2.18E-05
YLR069C	MEF1	-1.245	0.422	6.94E-04
YKR089C	TGL4	-1.245	0.422	1.39E-02
YMR173W	DDR48	-1.245	0.422	3.78E-06
YLR056W	ERG3	-1.243	0.422	1.24E-03
YOR044W	IRC23	-1.238	0.424	8.78E-06
YPR190C	RPC82	-1.235	0.425	9.76E-05
YKR024C	DBP7	-1.227	0.427	3.06E-06
YGR008C	STF2	-1.226	0.428	2.11E-03
YPL107W	YPL107W	-1.224	0.428	5.32E-05
YLR070C	XYL2	-1.221	0.429	1.82E-05
YEL020W-A	TIM9	-1.219	0.430	3.60E-04
YPL183C	RTT10	-1.216	0.431	2.13E-07
YDR184C	ATC1	-1.213	0.431	8.81E-04

YMR110C	HFD1	-1.212	0.432	5.15E-03
YGR209C	TRX2	-1.208	0.433	1.02E-02
YAL054C	ACS1	-1.207	0.433	1.15E-05
YHR160C	PEX18	-1.206	0.434	1.41E-02
YOL029C	YOL029C	-1.205	0.434	3.85E-07
YMR196W	YMR196W	-1.205	0.434	9.10E-03
YIL111W	COX5B	-1.202	0.435	4.32E-02
YAL008W	FUN14	-1.200	0.435	4.30E-05
YDR258C	HSP78	-1.194	0.437	7.45E-06
YCR045C	RRT12	-1.191	0.438	4.23E-04
YBR054W	YRO2	-1.191	0.438	1.52E-03
YPL068C	YPL068C	-1.186	0.439	2.15E-05
Q0075	AI5_BETA	-1.183	0.440	5.18E-03
YDR218C	SPR28	-1.182	0.441	9.86E-05
YGL101W	YGL101W	-1.179	0.442	5.95E-04
YDR527W	RBA50	-1.178	0.442	9.42E-05
YMR093W	UTP15	-1.177	0.442	1.13E-03
YHR087W	RTC3	-1.174	0.443	4.97E-03
YOL083W	ATG34	-1.173	0.443	1.25E-04
YIL125W	KGD1	-1.172	0.444	3.58E-04
YOL124C	TRM11	-1.170	0.444	1.32E-02
YLR295C	ATP14	-1.167	0.445	1.58E-04
YCL054W	SPB1	-1.165	0.446	3.90E-02
Q0080	ATP8	-1.161	0.447	1.17E-02
YFL034C-A	RPL22B	-1.160	0.448	1.09E-05
YGR239C	PEX21	-1.157	0.448	1.20E-03
YBR141C	BMT2	-1.155	0.449	1.59E-05
YGR266W	YGR266W	-1.155	0.449	1.39E-02
YER044C	ERG28	-1.154	0.449	1.57E-03
YMR229C	RRP5	-1.151	0.450	1.13E-05
YOR340C	RPA43	-1.144	0.452	2.93E-08
YJL010C	NOP9	-1.144	0.452	1.54E-05
YKL093W	MBR1	-1.144	0.453	7.48E-03
YOR187W	TUF1	-1.142	0.453	1.04E-05
YAL063C	FLO9	-1.141	0.453	2.61E-04
YDR465C	RMT2	-1.139	0.454	5.73E-03
YBL028C	YBL028C	-1.137	0.455	3.39E-03
YGR145W	ENP2	-1.134	0.456	1.05E-04
YPL117C	IDI1	-1.132	0.456	1.17E-03
YGR087C	PDC6	-1.131	0.457	5.93E-04
YDL054C	MCH1	-1.130	0.457	4.05E-02
YOR154W	SLP1	-1.129	0.457	9.53E-06
YGR021W	YGR021W	-1.121	0.460	5.93E-04
YFR011C	MIC19	-1.121	0.460	1.42E-05
YMR131C	RRB1	-1.120	0.460	1.44E-03
YHL026C	YHL026C	-1.114	0.462	3.13E-07

YOL122C	SMF1	-1.113	0.462	3.48E-03
YOR186W	YOR186W	-1.113	0.462	2.92E-05
YHR148W	IMP3	-1.111	0.463	1.32E-04
YOR360C	PDE2	-1.110	0.463	1.29E-06
YJL122W	ALB1	-1.110	0.463	3.62E-04
EC1118_1O4_6601g	EC1118_1O4_6601g	-1.110	0.463	1.94E-03
YLR326W	YLR326W	-1.107	0.464	7.61E-05
YJL108C	PRM10	-1.106	0.464	3.88E-03
YER182W	FMP10	-1.106	0.465	9.42E-06
YBR239C	ERT1	-1.105	0.465	1.10E-05
YPL043W	NOP4	-1.102	0.466	3.46E-02
YGR123C	PPT1	-1.094	0.468	4.70E-05
YIL039W	TED1	-1.090	0.470	2.05E-03
YLR203C	MSS51	-1.090	0.470	6.35E-03
YPL183W-A	RTC6	-1.088	0.470	1.37E-02
YDR248C	YDR248C	-1.086	0.471	2.28E-03
Q0250	COX2	-1.086	0.471	6.25E-03
YDL214C	PRR2	-1.084	0.472	9.60E-03
YBR172C	SMY2	-1.083	0.472	7.14E-04
YLR074C	BUD20	-1.081	0.473	4.29E-04
YAL039C	CYC3	-1.079	0.473	2.19E-04
YKR071C	DRE2	-1.075	0.475	3.50E-04
YNL002C	RLP7	-1.071	0.476	7.79E-03
YIL091C	UTP25	-1.070	0.476	1.44E-03
YBR111C	YSA1	-1.067	0.477	2.59E-04
YKR099W	BAS1	-1.062	0.479	4.17E-03
YKL195W	MIA40	-1.060	0.480	1.89E-05
YHR092C	HXT4	-1.060	0.480	2.71E-03
YLR153C	ACS2	-1.056	0.481	7.62E-04
YMR049C	ERB1	-1.056	0.481	5.73E-04
YKL094W	YJU3	-1.055	0.481	4.60E-04
YPL104W	MSD1	-1.054	0.482	1.42E-04
YOL028C	YAP7	-1.054	0.482	4.69E-04
YML028W	TSA1	-1.049	0.483	8.31E-04
YHR170W	NMD3	-1.048	0.484	6.99E-06
YOR161C	PNS1	-1.046	0.484	2.98E-04
YOR206W	NOC2	-1.046	0.484	4.52E-06
YOR095C	RKI1	-1.045	0.485	9.40E-04
YKL151C	YKL151C	-1.042	0.486	1.25E-03
YGL001C	ERG26	-1.038	0.487	4.61E-03
YFL036W	RPO41	-1.037	0.487	8.00E-05
YDR178W	SDH4	-1.035	0.488	2.61E-02
YIL155C	GUT2	-1.033	0.489	4.14E-04
YLR023C	IZH3	-1.033	0.489	1.41E-02
YJL185C	ATG36	-1.032	0.489	9.56E-04
YGR102C	GTF1	-1.032	0.489	5.00E-03

YNL115C	YNL115C	-1.027	0.491	1.72E-04
YJL045W	YJL045W	-1.027	0.491	1.97E-03
YGR255C	COQ6	-1.024	0.492	5.15E-04
YPL172C	COX10	-1.020	0.493	1.19E-04
YML122C	YML122C	-1.010	0.497	2.42E-03
YGR229C	SMI1	-1.010	0.497	4.97E-03
YML076C	WAR1	-1.008	0.497	1.44E-03
YMR089C	YTA12	-1.008	0.497	3.33E-02
YNL306W	MRPS18	-1.007	0.497	6.97E-04
YNL186W	UBP10	-1.007	0.498	8.48E-04
YBR114W	RAD16	-1.007	0.498	1.97E-03
YBR155W	CNS1	-1.006	0.498	7.16E-06
YDL150W	RPC53	-1.004	0.499	1.41E-02
YHR077C	NMD2	-1.004	0.499	6.84E-04
YNL117W	MLS1	-1.004	0.499	3.21E-03
YER045C	ACA1	-1.003	0.499	2.26E-03
YLR080W	EMP46	-1.001	0.500	3.36E-05
YMR220W	ERG8	-1.000	0.500	2.60E-03
YGR060W	ERG25	-0.998	0.501	7.76E-03
YPL146C	NOP53	-0.996	0.501	2.17E-03
YML008C	ERG6	-0.995	0.502	3.01E-04
EC1118_1O4_6579g	EC1118_1O4_6579g	-0.995	0.502	4.54E-03
YDR120C	TRM1	-0.990	0.503	7.29E-04
YNL173C	MDG1	-0.988	0.504	8.38E-06
YHL029C	OCA5	-0.984	0.506	2.60E-06
YML081C-A	ATP18	-0.983	0.506	1.68E-04
YOL010W	RCL1	-0.982	0.506	2.70E-02
YMR037C	MSN2	-0.982	0.506	2.25E-04
YML100W	TSL1	-0.981	0.507	1.28E-02
YLR064W	PER33	-0.980	0.507	3.64E-04
YPL207W	TYW1	-0.980	0.507	2.33E-05
YBL099W	ATP1	-0.977	0.508	4.12E-02
YIL096C	BMT5	-0.976	0.509	2.11E-02
YHR055C	CUP1-2	-0.976	0.509	4.57E-03
YKL148C	SDH1	-0.975	0.509	5.33E-03
YKR081C	RPF2	-0.974	0.509	4.67E-04
YMR271C	URA10	-0.974	0.509	1.74E-02
YPL198W	RPL7B	-0.972	0.510	2.96E-03
YPL012W	RRP12	-0.972	0.510	4.58E-05
YHR052W	CIC1	-0.968	0.511	5.23E-04
YKL085W	MDH1	-0.967	0.511	2.92E-05
YLR051C	FCF2	-0.963	0.513	6.83E-04
YBR169C	SSE2	-0.961	0.514	2.38E-02
YPL170W	DAP1	-0.956	0.515	4.99E-03
YNL308C	KRI1	-0.955	0.516	7.45E-04
YLR196W	PWP1	-0.955	0.516	6.62E-04

YJR080C	AIM24	-0.947	0.519	8.00E-05
YML072C	TCB3	-0.945	0.519	1.11E-03
YLR218C	COA4	-0.944	0.520	2.32E-02
YHR106W	TRR2	-0.942	0.520	2.38E-04
YPR192W	AQY1	-0.942	0.521	3.81E-03
YKL194C	MST1	-0.941	0.521	2.59E-03
YOR100C	CRC1	-0.941	0.521	6.23E-03
YDL209C	CWC2	-0.940	0.521	2.02E-03
YGR035C	YGR035C	-0.939	0.522	1.79E-03
YPR026W	ATH1	-0.938	0.522	1.30E-02
YNL062C	GCD10	-0.936	0.523	2.11E-04
YML123C	PHO84	-0.935	0.523	1.09E-04
YNR001C	CIT1	-0.934	0.523	4.64E-02
YNR054C	ESF2	-0.933	0.524	6.74E-03
YMR268C	PRP24	-0.932	0.524	2.43E-04
YJR121W	ATP2	-0.930	0.525	4.37E-03
YLL023C	POM33	-0.927	0.526	3.28E-02
YPL026C	SKS1	-0.927	0.526	7.54E-03
YOR185C	GSP2	-0.924	0.527	2.63E-02
YFR048W	RMD8	-0.922	0.528	6.10E-05
YGL029W	CGR1	-0.921	0.528	2.66E-03
YLL034C	RIX7	-0.920	0.529	3.01E-04
YBL098W	BNA4	-0.919	0.529	3.03E-03
YOR252W	TMA16	-0.918	0.529	2.94E-05
YJL062W-A	COA3	-0.918	0.529	1.41E-03
YOR119C	RIO1	-0.917	0.530	4.52E-02
YNL156C	NSG2	-0.917	0.530	2.11E-03
YNL124W	NAF1	-0.914	0.531	1.82E-05
YBR021W	FUR4	-0.914	0.531	1.13E-03
YDR449C	UTP6	-0.912	0.531	3.02E-03
YJR119C	JHD2	-0.910	0.532	5.12E-05
YGR245C	SDA1	-0.910	0.532	3.44E-02
YLR256W	HAP1	-0.909	0.532	9.02E-03
YBL039C	URA7	-0.908	0.533	1.45E-02
YDR048C	YDR048C	-0.906	0.534	3.39E-02
YBR096W	YBR096W	-0.905	0.534	4.41E-05
YKL143W	LTV1	-0.904	0.535	5.41E-05
YGR053C	YGR053C	-0.904	0.535	6.13E-04
YER150W	SPI1	-0.903	0.535	1.04E-03
Q0060	AI3	-0.903	0.535	1.35E-02
YDR031W	MIX14	-0.902	0.535	6.49E-04
YPL093W	NOG1	-0.902	0.535	1.16E-04
YFR025C	HIS2	0.904	1.871	5.70E-03
YIL173W	VTH1	0.905	1.873	1.09E-02
YLR179C	YLR179C	0.909	1.878	9.15E-03
YDL059C	RAD59	0.913	1.883	5.41E-05

YPL267W	ACM1	0.915	1.886	4.32E-02
YIR034C	LYS1	0.918	1.890	3.39E-05
YPL031C	PHO85	0.920	1.892	2.77E-02
YBR243C	ALG7	0.920	1.893	2.11E-03
YKL127W	PGM1	0.927	1.901	5.32E-05
YDR261C	EXG2	0.929	1.904	2.03E-02
YER032W	FIR1	0.929	1.905	1.29E-03
YDR487C	RIB3	0.931	1.906	7.76E-03
YDR541C	YDR541C	0.933	1.909	1.67E-04
YBR071W	YBR071W	0.936	1.913	1.24E-02
YIL123W	SIM1	0.937	1.914	4.27E-02
YCL055W	KAR4	0.938	1.915	3.62E-05
EC1118_1F14_0133g	EC1118_1F14_0133g	0.940	1.918	5.50E-05
YKL213C	DOA1	0.940	1.918	4.32E-05
YGL256W	ADH4	0.943	1.923	5.73E-03
YCL030C	HIS4	0.945	1.926	3.76E-03
YBL082C	ALG3	0.947	1.928	6.14E-03
YPR105C	COG4	0.951	1.933	9.96E-03
EC1118_1O4_6491g	EC1118_1O4_6491g	0.954	1.937	9.16E-04
YOR202W	HIS3	0.961	1.947	5.43E-03
YOR094W	ARF3	0.971	1.960	1.47E-05
YMR189W	GCV2	0.975	1.965	6.70E-04
YIR028W	DAL4	0.979	1.971	7.93E-05
YNL253W	TEX1	0.982	1.975	6.46E-03
YHR213W	YHR213W	0.983	1.976	1.13E-02
YMR120C	ADE17	0.990	1.986	6.68E-03
YOR315W	SFG1	0.992	1.989	1.58E-02
YDR408C	ADE8	0.994	1.992	2.68E-03
YOR026W	BUB3	1.001	2.002	2.90E-03
YPL156C	PRM4	1.003	2.004	1.03E-02
YAR023C	YAR023C	1.008	2.012	4.08E-05
YEL040W	UTR2	1.013	2.018	1.44E-03
YHR094C	HXT1	1.014	2.020	1.52E-02
YKL126W	YPK1	1.016	2.023	6.62E-04
YGL021W	ALK1	1.025	2.034	4.36E-03
YDL010W	GRX6	1.025	2.035	1.35E-03
YER019W	ISC1	1.026	2.036	1.08E-03
EC1118_1F14_0100g	EC1118_1F14_0100g	1.029	2.040	3.11E-05
YFR030W	MET10	1.029	2.041	2.33E-04
YNR059W	MNT4	1.034	2.048	3.28E-03
YNL128W	TEP1	1.042	2.059	2.09E-02
YCR098C	GIT1	1.044	2.062	1.41E-03
YOR326W	MYO2	1.045	2.063	3.93E-03
YJL158C	CIS3	1.045	2.064	7.20E-03
YJL060W	BNA3	1.045	2.064	1.62E-02
YEL058W	PCM1	1.051	2.072	5.33E-03

YMR161W	HLJ1	1.053	2.075	3.24E-02
YNL331C	AAD14	1.056	2.079	1.60E-03
YHR041C	SRB2	1.059	2.083	2.82E-04
YDR400W	URH1	1.060	2.085	2.28E-04
YNL312W	RFA2	1.061	2.087	6.97E-04
YKR019C	IRS4	1.067	2.095	1.04E-03
YPL214C	THI6	1.070	2.099	3.04E-06
YER136W	GDI1	1.073	2.104	2.26E-03
YDR089W	YDR089W	1.077	2.110	1.22E-04
YDL238C	GUD1	1.087	2.124	2.33E-05
YKR039W	GAP1	1.088	2.125	1.76E-03
EC1118_1F14_0111g	EC1118_1F14_0111g	1.092	2.131	2.01E-02
YLR126C	YLR126C	1.093	2.133	2.52E-03
YML124C	TUB3	1.099	2.143	1.42E-04
YBR298C	MAL31	1.101	2.145	2.67E-04
YEL066W	HPA3	1.103	2.148	1.35E-04
YLR034C	SMF3	1.103	2.148	8.23E-03
YDR380W	ARO10	1.104	2.150	4.97E-03
YIL116W	HIS5	1.109	2.157	6.59E-04
YOR248W	YOR248W	1.127	2.185	2.11E-02
YOR184W	SER1	1.130	2.189	1.23E-04
YPR167C	MET16	1.131	2.190	8.26E-06
YDL012C	YDL012C	1.133	2.194	1.25E-02
YLR194C	YLR194C	1.135	2.196	1.23E-03
YFL051C	YFL051C	1.139	2.202	1.60E-03
YKL073W	LHS1	1.142	2.207	1.51E-02
YDL215C	GDH2	1.148	2.216	1.85E-04
YIR029W	DAL2	1.155	2.227	1.85E-05
YNR050C	LYS9	1.156	2.229	5.33E-03
YCL034W	LSB5	1.174	2.256	1.24E-02
YKR091W	SRL3	1.190	2.281	3.37E-03
YBR145W	ADH5	1.190	2.282	1.15E-02
YER060W-A	FCY22	1.194	2.289	3.25E-04
YOR237W	HES1	1.209	2.311	1.07E-03
YOR247W	SRL1	1.218	2.326	7.52E-05
YFL010C	WWM1	1.219	2.327	1.41E-03
YCL064C	CHA1	1.224	2.336	9.47E-03
YHL048W	COS8	1.227	2.341	1.84E-03
YJR137C	MET5	1.229	2.344	6.04E-05
YOL016C	CMK2	1.247	2.374	2.28E-02
EC1118_1O30_0012g	EC1118_1O30_0012g	1.259	2.394	2.27E-03
YNR068C	YNR068C	1.262	2.398	1.18E-04
YNL333W	SNZ2	1.271	2.413	3.76E-06
YFR056C	YFR056C	1.276	2.422	9.56E-05
YOR192C	THI72	1.296	2.455	2.78E-06
YGL224C	SDT1	1.298	2.460	2.45E-03

YOR321W	PMT3	1.302	2.466	9.56E-05
YFL059W	SNZ3	1.326	2.507	7.00E-05
YDR354W	TRP4	1.333	2.520	1.94E-02
YOR222W	ODC2	1.354	2.556	8.28E-06
YCR020C	PET18	1.363	2.573	2.53E-03
YGL179C	TOS3	1.364	2.574	1.32E-07
YMR020W	FMS1	1.371	2.586	4.01E-04
YBR162C	TOS1	1.373	2.591	3.86E-02
YJR148W	BAT2	1.388	2.617	7.43E-05
YOR385W	YOR385W	1.406	2.650	5.18E-03
YNL024C	YNL024C	1.407	2.652	1.57E-02
YDR213W	UPC2	1.426	2.687	1.69E-03
YGL202W	ARO8	1.461	2.754	4.52E-02
YMR305C	SCW10	1.464	2.759	2.29E-02
YLR257W	YLR257W	1.483	2.795	9.46E-04
YOL064C	MET22	1.494	2.817	1.52E-03
YOL154W	ZPS1	1.508	2.844	3.66E-05
YBR040W	FIG1	1.563	2.954	4.08E-03
YOL116W	MSN1	1.574	2.978	1.37E-04
YMR304C-A	YMR304C-A	1.599	3.029	1.44E-05
YDL239C	ADY3	1.604	3.041	1.25E-04
YKR013W	PRY2	1.627	3.089	3.90E-06
YFL021W	GAT1	1.628	3.090	3.25E-06
YJL088W	ARG3	1.638	3.111	1.70E-03
YEL065W	SIT1	1.656	3.152	3.34E-05
YIR030C	DCG1	1.663	3.166	1.04E-06
YCR060W	TAH1	1.668	3.179	4.22E-02
YLR089C	ALT1	1.670	3.183	5.16E-07
YIR032C	DAL3	1.691	3.228	6.83E-04
YGL253W	HXK2	1.714	3.281	2.33E-05
YOL058W	ARG1	1.741	3.343	1.59E-04
YPL258C	THI21	1.746	3.355	2.78E-03
YDR090C	YDR090C	1.799	3.479	9.22E-03
YKR053C	YSR3	1.799	3.480	7.87E-04
YGL089C	MF(ALPHA)2	1.819	3.528	7.96E-04
YPR194C	OPT2	1.843	3.587	4.20E-04
YJR152W	DAL5	1.848	3.601	1.32E-07
YDR242W	AMD2	1.899	3.730	2.88E-04
YNL142W	MEP2	1.919	3.781	7.79E-06
YMR088C	VBA1	1.931	3.814	1.10E-06
EC1118_1O4_6513g	EC1118_1O4_6513g	2.099	4.284	1.48E-05
YOL019W	YOL019W	2.140	4.409	5.22E-03
YER185W	PUG1	2.142	4.413	2.80E-03
YNL129W	NRK1	2.192	4.568	4.90E-05
YDL145C	COP1	2.261	4.795	1.10E-03
YJL174W	KRE9	2.311	4.963	3.85E-02

EC1118_1O4_6502g	EC1118_1O4_6502g	2.349	5.094	2.70E-02
YIL165C	YIL165C	2.378	5.199	3.65E-06
YKR034W	DAL80	2.414	5.331	2.33E-05
YIL164C	NIT1	2.459	5.497	2.12E-09
YGR190C	YGR190C	2.466	5.524	1.19E-02
YIR031C	DAL7	2.559	5.892	1.82E-05
YHR029C	YHI9	2.566	5.921	1.58E-07
YJR151C	DAN4	2.778	6.859	6.21E-08
YJL172W	CPS1	3.167	8.981	6.21E-08
YCR105W	ADH7	3.188	9.112	1.56E-06
YFR055W	IRC7	3.404	10.584	1.98E-02
YPL265W	DIP5	3.519	11.464	1.42E-04
YMR317W	YMR317W	4.361	20.554	2.51E-12
